# On-surface synthesis of a nitrogen-embedded buckybowl with inverse Stone–Thrower–Wales topology

**DOI:** 10.1038/s41467-018-04144-5

**Published:** 2018-04-30

**Authors:** Shantanu Mishra, Maciej Krzeszewski, Carlo A. Pignedoli, Pascal Ruffieux, Roman Fasel, Daniel T. Gryko

**Affiliations:** 1 Empa, Swiss Federal Laboratories for Materials Science and Technology, Überlandstrasse 129, Dübendorf, 8600 Switzerland; 2 Institute of Organic Chemistry, Polish Academy of Sciences, Kasprzaka 44-52, Warsaw, 01-224 Poland; 3 Department of Chemistry and Biochemistry, University of Bern, Freiestrasse 3, Bern, 3012 Switzerland

## Abstract

Curved π-conjugated polycyclic aromatic hydrocarbons, buckybowls, constitute an important class of materials with wide applications in materials science. Heteroatom doping of buckybowls is a viable route to tune their intrinsic physicochemical properties. However, synthesis of heteroatom-doped buckybowls is a challenging task. We report on a combined in-solution and on-surface synthetic strategy toward the fabrication of a buckybowl containing two fused nitrogen-doped pentagonal rings. We employ ultra-high-resolution scanning tunneling microscopy and spectroscopy, in combination with density functional theory calculations to characterize the final compound. The buckybowl contains a unique combination of non-hexagonal rings at its core, identified as the inverse Stone–Thrower–Wales topology, resulting in a distinctive bowl-opening-down conformation of the buckybowl on the surface. Our controlled design of non-alternant, heteroatom-doped polycyclic aromatic frameworks with established bottom-up fabrication techniques opens new opportunities in the synthesis of carbon nanostructures with the perspective of engineering properties of graphene-based devices.

## Abstract (web summary)

Heteroatom doping of buckybowls is a viable route to tune their intrinsic physico-chemical properties, but their synthesis remains challenging. Here, the authors report on a combined in-solution and on-surface synthetic strategy towards the fabrication of a buckybowl containing two fused nitrogen-doped pentagonal rings.

## Introduction

Polycyclic aromatic hydrocarbons (PAHs) and their heterocyclic analogs possessing curved π-conjugated architectures (e.g., saddle- or bowl-shaped) attract substantial attention of the broad scientific community due to their unparalleled properties and potential application in the field of organic electronics^1–6^. Seminal publications on the synthesis of corannulene reported by Scott^7^ and Siegel^8,9^ encouraged chemists all over the world to pursue the goal of bottom-up synthesis of fullerene and carbon nanotubes^10^. Indeed, truly enthralling architectures have been synthesized over the recent years^11–14^. Displacement from planarity in π-extended systems usually requires harsh reaction conditions, which heterocycles rarely tolerate. Incorporation of heteroatoms into polycyclic aromatic frameworks, however, should drastically change their intrinsic optoelectronic properties^15–18^. Synthesis of nitrogen-embedded corannulene analogs is a challenging task. Only recently, two groups independently reported preparation of such conjugates^19,20^.

Serendipitous discovery made in our laboratory in 2013 on the one-pot, multicomponent synthesis for 1,2,4,5-tetraarylpyrrolo[3,2-*b*]pyrroles (TAPPs) opens up the possibilities for the formation of previously unknown π-expanded aza-analogs of PAHs^21–23^. A particularly suitable feature of this methodology is the fact that sterically congested *ortho*-substituted benzaldehydes give products in high yields, which makes this approach a powerful tool for the construction of highly conjugated molecules comprising pyrrolo[3,2-*b*]pyrrole (PP) units^24–28^. Conversely, presence of two fused pentagonal rings in the center makes the pursuit of circularly conjugated species particularly complex, since it requires thermodynamically demanding closure of two heptagonal rings. In this regard, modern on-surface chemistry serves as a powerful synthetic tool toward bottom-up fabrication of complex nanostructures unattainable via solution chemistry^29^. Many challenging syntheses of carbon-based nanostructures have been realized through this approach including atomically precise graphene nanoribbons^30^, single-chirality single-walled carbon nanotubes^31^, longest to-date acenes^32^ and periacenes^33^, and embedment of non-hexagonal rings in PAHs^34^.

Here we report a concise preparation of the first fully conjugated buckybowl based on the PP scaffold. Our synthetic pathway encompasses a combination of in-solution and on-surface chemistry. In particular, on-surface cyclodehydrogenation of suitably designed precursor molecules on a Au(111) surface was the crucial step toward formation of the nitrogen-embedded buckybowl. Bond-resolved scanning tunneling microscopy (STM) unequivocally confirms the structure of the final molecule. In addition, scanning tunneling spectroscopy (STS) in combination with density functional theory (DFT) calculations further lend support to the synthesis of the final product. Notably, the core of the buckybowl contains a unique combination of pentagonal and heptagonal rings: the inverse Stone–Thrower–Wales (ISTW) topology, a topological defect in graphene that remains challenging to obtain experimentally. As a consequence of this unique topology, the nitrogen-doped buckybowl adopts a dome-shaped bowl-opening-down conformation on the surface—a property unprecedented for isolated buckybowls on surface.

## Results

### Design and synthesis of precursor molecules

Our previous studies indicated that scrupulous design of TAPPs bearing bromine atoms and aryl rings in *ortho* position with respect to the central core facilitates smooth expansion of the π-system by means of intramolecular direct arylation and oxidative aromatic coupling, respectively^25,27^. Highly conjugated molecules based on the PP core adopt nonplanar architecture due to the close spatial vicinity of adjacent benzene rings.

**Fig. 1:**
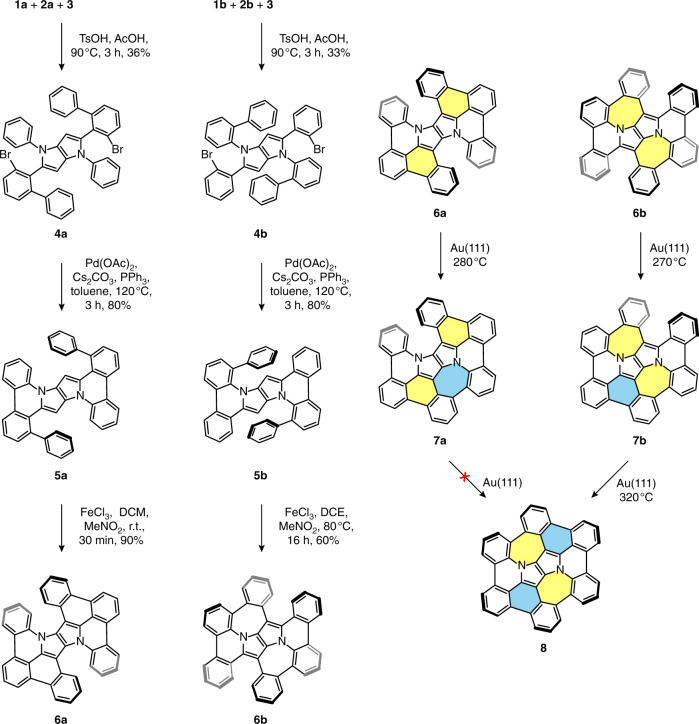
Synthetic route to the nitrogen-embedded buckybowl **8**. (Left panel) Solution-synthetic routes to precursor molecules **6a** and **6b**. (Right panel) Subsequent on-surface synthetic route to partially closed species **7a**, **7b** and the fully conjugated nitrogen-embedded buckybowl **8**. Crucial rings formed in the final steps are marked in colors (via oxidative aromatic coupling—yellow, via on-surface cyclodehydrogenation—blue)

Our initial assumption was that the naked analog **6a** (lacking any bulky groups at the peripheries) might serve as a precursor for the on-surface cyclodehydrogenation. We thus began with the synthesis of the parent TAPP **4a** bearing bromine atoms and phenyl rings in suitable *ortho* positions with respect to the central core (Fig. 1, left panel). One-pot multicomponent condensation between 2-bromo-6-phenylbenzaldehyde (**1a**), aniline (**2a**) and butane-2,3-dione (**3**) gives **4a** in 36% yield. Subjecting **4a** to palladium-catalyzed intramolecular direct arylation leads smoothly to the formation of two new carbon–carbon bonds around the core and hence to the planar derivative **5a** obtained in 80% yield. In the final step we employed oxidative aromatic coupling utilizing iron(III) chloride. As a result, the central core is connected with two peripheral benzene rings leading to the nonplanar compound **6a** produced in 90% yield. Subjecting **6a** prepared in this way to subsequent traditional oxidation methods, such as DDQ/TfOH, MoCl_5_, etc., however, does not give the desired compound **8**.

Previous success in the formation of highly π-extended species by surface-assisted techniques motivated us to apply this approach to compound **6a**. In this case, on-surface cyclodehydrogenation effectively leads to the formation of half-closed species with only one heptagonal ring, **7a** (Fig. 1, right panel). Conversely, the targeted fully conjugated compound containing two heptagons is not formed.

This partial success prompted us to design an alternative precursor **6b**, which possesses two preformed heptagonal rings. In this approach, additional benzene rings originate from aniline derivatives. Indeed, condensation between 2-bromobenzaldehyde (**1b**), 2-aminobiphenyl (**2b**), and butane-2,3-dione (**3**) gives TAPP **4b** in 33% yield (Fig. 1, left panel). Analogous to the previous synthesis, subjecting TAPP **4b** to palladium-catalyzed intramolecular direct arylation produces planar π-extended derivative **5b** in 80% yield. Compound **5b** undergoes efficient oxidation with iron(III) chloride in dichloroethane at 80 °C giving **6b** in 60% yield. This reaction leads to the formation of two new C–C bonds and results in a highly distorted π-extended PP derivative possessing two heptagons in its structure.

Unlike **6a**, cyclodehydrogenation of **6b** requires the closure of two hexagonal instead of two heptagonal rings to obtain the fully conjugated target species **8**. Opportunely, precursor **6b** proves to be viable for on-surface cyclodehydrogenation. Annealing this compound to 320 °C under ultra-high vacuum (UHV) conditions on a Au(111) substrate effectively leads to the formation of the desired nitrogen-embedded buckybowl **8** (Fig. 1, right panel).

### On-surface synthesis of nitrogen-embedded buckybowls

**Fig. 2:**
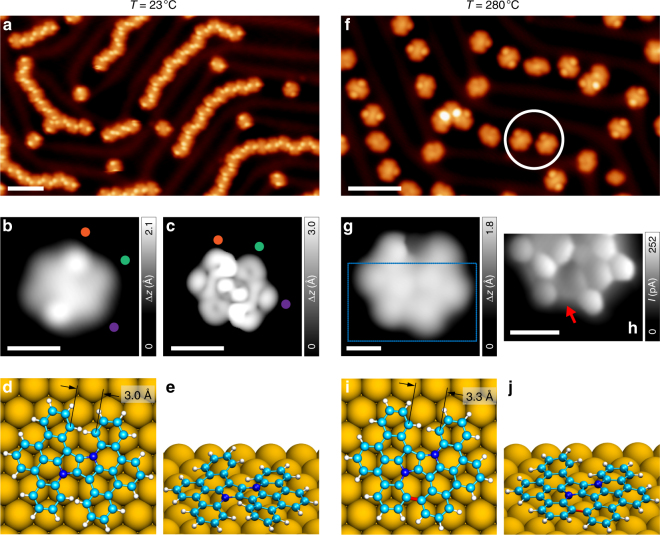
On-surface reaction of **6a** on Au(111). **a** Overview STM topography image of the surface after room temperature deposition of **6a**, revealing the coexistence of predominant chains running along *fcc* domains, and isolated molecules (*V* = −1 V, *I* = 10 pA, scale bar: 5 nm). **b** High-resolution STM image (*V* = −0.1 V, *I* = 50 pA, scale bar: 1 nm) and **c** DFT-simulated STM image (*V* = −0.2 V, scale bar: 1 nm) of **6a**. The colored dots serve as a guide to the eye for comparing the intramolecular features in experimental and simulated STM images. **d**, **e** Top and side views of the DFT equilibrium geometry of **6a**. **f** Overview STM topography image of the surface after annealing to 280 °C (*V* = −1 V, *I* = 10 pA, scale bar: 5 nm). The white circle highlights the half-closed species **7a**, presenting only one lobe. **g** High-resolution STM image of **7a** acquired with a CO tip (*V* = 10 mV, *I* = 50 pA, scale bar: 0.5 nm). The blue rectangle marks the acquisition area of the UHR-STM image. **h** Corresponding UHR-STM image of **7a** (open feedback parameters: *V* = 5 mV, *I* = 50 pA; Δ*z* = −50 pm; scale bar: 0.5 nm). The red arrow indicates the heptagonal ring formed after this annealing step. **i**, **j** Top and side views of the DFT equilibrium geometry of **7a**. The newly formed bond at this annealing step is highlighted in red

After sublimation of precursor **6a** under UHV conditions onto an atomically clean Au(111) surface held at room temperature, large-scale STM images (Fig. 2a) acquired at 5 K show the presence of self-assembled chains that follow the Au(111) herringbone reconstruction, along with sporadically distributed individual molecules. Figure 2b shows a representative high-resolution STM image of the predominant species on the surface (95% of 170 molecules), which presents two bright lobes of 2.5 Å in height (measured at a sample bias of −1 V). The experimental features are well reproduced by the DFT-optimized geometry of **6a** on a Au(111) surface (Fig. 2d, e), which shows that **6a** adopts a twisted conformation on the surface. In addition, the corresponding STM simulation (Fig. 2c) agrees well with the experimental STM image.

Annealing the surface to 280 °C brings about notable changes in the surface topography. Large-scale STM images (Fig. 2f) show an absence of chains, with isolated molecules present on the surface. Majority of the species have a two-lobed appearance (38% of 100 molecules) and presumably did not undergo cyclodehydrogenation. Notably, there is appearance of a new species (15% of 100 molecules) that present only one lobe of much lower apparent height compared to **6a** (highlighted with a white circle in Fig. 2f). While it could be speculated that appearance of only one lobe hints at partial intramolecular cyclodehydrogenation and concomitant planarization of one lobe, it is rather difficult to conclusively identify the exact chemical structure of this product via STM imaging alone, which fundamentally probes the local density of states (LDOS) near the Fermi energy. To circumvent this limitation, we functionalize our tip by a CO molecule that helps us probe the sample in the regime of the onset of Pauli repulsion, thereby allowing to visualize the chemical structure of the molecule. It has been shown that STM tips functionalized with single atoms or molecules can serve as a nanoscale transducer, converting atomic-scale forces exerted by the sample on the tip functional moiety to conductance variations in the tunneling junction^35,36^. Our choice of CO as the tip functional moiety is governed by its facile manipulation on metal or insulating substrates and its rigid bonding to the metal tip, yielding images of high fidelity^36^. Figure 2g shows a high-resolution STM image of the one-lobed species acquired with a CO-functionalized tip. While only weak intramolecular contrast can be discerned, it is nevertheless clear that this species adsorbs flat on the surface except for a sharp ridge around the position of the lobe. Furthermore, at the expected position of the second lobe in **6a**, we instead find a smooth bay indicating a possible bond closure. Finally, an ultra-high-resolution STM (UHR-STM) image (Fig. 2h) of the bottom part of the molecule (highlighted with a blue rectangle in Fig. 2g) clearly shows formation of a heptagonal ring at the aforementioned bay position (indicated with a red arrow), which leads us to conclude that at this temperature, partial intramolecular cyclodehydrogenation occurs to form the half-closed species **7a**. Figure 2i, j show the DFT-optimized structure of **7a** on Au(111), which correctly reproduces the experimental features—i.e., the out-of-plane bending of a benzenoid ring corresponding to a single lobe in the STM images, and the otherwise planar adsorption geometry of the molecule on the surface.

Finally, we anneal the surface upwards of 350 °C in an attempt to induce full ring closure. Unfortunately, it was seen that for higher temperatures, intermolecular covalent coupling and side reactions dominate and full ring closure is not observed (see Supplementary Fig. 21). It is worth to mention that we systematically tried to induce ring closure on more catalytically active coinage metal surfaces—i.e., Ag(111) and Cu(111). On Ag(111) (see Supplementary Fig. 19) half-closure to form **7a** does take place, but the molecules start to decompose upwards of 300 °C without full ring closure. On the most active Cu(111) surface (see Supplementary Fig. 20), we observe neither half- nor fully-closed species, with widespread intermolecular coupling and decomposition upwards of 260 °C. Thus, with precursor **6a**, while intramolecular cyclodehydrogenation to form one heptagonal ring is feasible (on Au(111) and Ag(111) surfaces), closure of the second heptagonal ring, however, is not favored.

**Fig. 3:**
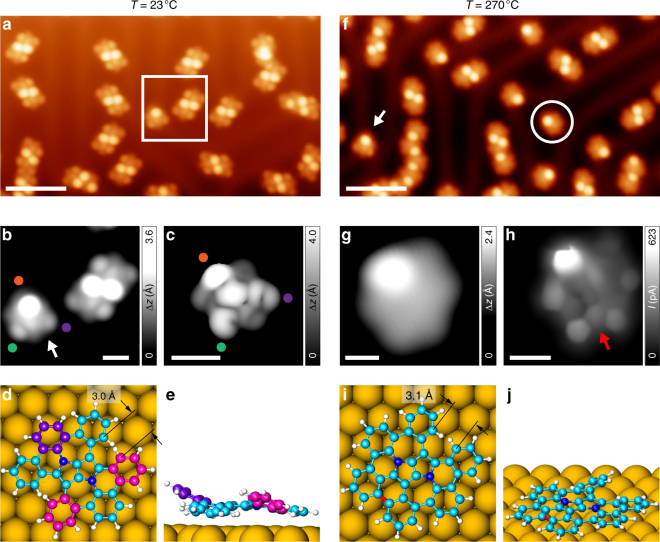
On-surface reaction of **6b** on Au(111). **a** Overview STM topography image of the surface after room temperature deposition of **6b**, showing the predominant presence of self-assembled dimers (*V* = −1 V, I = 50 pA, scale bar: 5 nm). **b** High-resolution STM image of the highlighted area in **a**, consisting of a dimer, and an isolated molecule indicated with the white arrow (*V* = −1 V, *I* = 70 pA, scale bar: 1 nm). **c** DFT-simulated STM image of **6b** corroborating the experimentally observed features (*V* = −1 V, scale bar: 1 nm). The colored dots serve as a guide to the eye for comparing the intramolecular features in experimental and simulated STM images. **d**, **e** Top and side views of the DFT equilibrium geometry of **6b**. Rings highlighted in violet and magenta colors show an out-of-plane bending contributing to the distinguishing features in the STM images. **f** Overview STM topography image of the surface after annealing to 270 °C (*V* = −1 V, *I* = 40 pA, scale bar: 5 nm). The white circle highlights the half-closed species **7b**. The white arrow indicates a still-present **6b** on the surface. **g** High-resolution STM image of **7b** (*V* = 10 mV, *I* = 50 pA, scale bar: 0.5 nm). **h** Corresponding UHR-STM image of **7b** (open feedback parameters: *V* = −5 mV, *I* = 50 pA; Δ*z* = −40 pm; scale bar: 0.5 nm). The red arrow indicates the hexagonal ring formed after this annealing step. **i**, **j** Top and side views of the DFT equilibrium geometry of **7b**. The newly formed bond at this annealing step is highlighted in red

We next investigate the on-surface reaction of an alternative precursor **6b**, which requires closure of two hexagonal rings (instead of two heptagonal rings as in **6a**), with preformed heptagonal rings. When **6b** is sublimed under UHV conditions onto an atomically clean Au(111) surface held at room temperature, large-scale STM images (Fig. 3a) show presence of predominantly discrete quasi-rectangular structures with two centrally located bright lobes of roughly 3.5 Å in height (measured at a sample bias of −1 V), in contrast to the long chains seen in case of **6a** (c.f. Fig. 2a). High-resolution STM imaging (Fig. 3b) reveals the intramolecular features in more detail, where it is seen that apart from the two prominent central lobes, additional lobes of weaker contrast are present at the terminus. In addition, we also find the occasional species with only one prominent lobe on one side and two lobes of weaker contrast on the opposite side (indicated with the white arrow in Fig. 3b). Evidently, the quasi-rectangular species are, in fact, non-covalently bonded, self-assembled dimers of the isolated species which we assign to be **6b**. The rather nontrivial conformation of **6b** presenting only one prominent lobe is elucidated by theoretical calculations, wherein its DFT-optimized structure (Fig. 3d, e) on Au(111) shows that, unlike **6a**, **6b** adopts neither twisted nor folded conformation, but a peculiar conformation in which two benzenoid rings are bent out-of-plane as in a folded conformation (magenta rings in Fig. 3d, e), giving rise to the two weak lobes in STM images; while one of the terminal benzenoid rings is strongly bent out-of-plane (violet ring in Fig. 3d, e), leading to the appearance of one prominent lobe in STM images. Furthermore, the STM image simulated for this conformation (Fig. 3c) agrees well with the experimental one, concluding the structural assignment of **6b** on the Au(111) surface.

Having clarified the structure of **6b**, we anneal the sample to 270 °C. Figure 3f shows a large-scale STM image of the surface after annealing, where there is a considerable decrease in the number of dimers with a concomitant increase in individual species presenting one prominent lobe (highlighted with a white circle in Fig. 3f). STM images show that these one-lobed species (referred to as **7b**), however, are different from **6b**, evidenced by a lack of two weaker lobes present at the molecule terminus. Whether isolated or present as dimers, **7b** constitutes the predominant species on surface (82% of 141 molecules), while only 7% is present as **6b** (the white arrow in Fig. 3f indicates an isolated **6b**). Figure 3g, h show a high-resolution STM image and the corresponding UHR-STM image of **7b**, respectively. Formation of a hexagonal ring (indicated with a red arrow) is evident in Fig. 3h, allowing us to conclude that **7b** results from partial intramolecular cyclodehydrogenation of **6b**, where one of the two hexagonal rings have formed. Its DFT-optimized structure on Au(111) (Fig. 3i, j) is in line with experimental observations, where the out-of-plane bent benzenoid ring is responsible for the bright lobe in STM/UHR-STM images, while the rest of the molecule adopts an almost planar geometry on the surface.

**Fig. 4:**
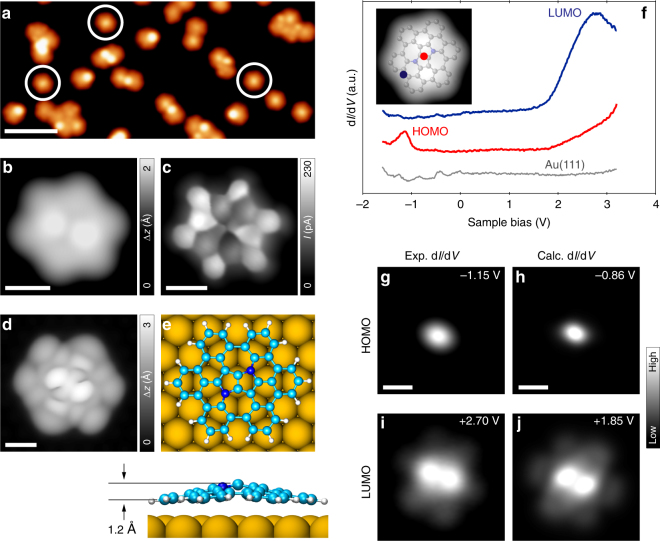
Structural and electronic properties of the nitrogen-embedded buckybowl **8**. **a** Overview STM topography image of the surface after an annealing step to 320 °C, showing the presence of mostly half-closed species (**7b**), and the fully closed buckybowls (**8**) highlighted by the white circles (*V* = −1.2 V, *I* = 20 pA, scale bar: 5 nm). **b** High-resolution STM image of **8** acquired with a CO tip (*V* = −0.1 V, *I* = 50 pA, scale bar: 0.5 nm). **c** Corresponding UHR-STM image of **8**, evidencing complete ring closure and presence of the ISTW topology at the core (open feedback parameters: *V* = 5 mV, *I* = 50 pA; Δ*z* = −50 pm; scale bar: 0.5 nm). **d** DFT-simulated STM image of **8** (*V* = −0.3 V, scale bar: 0.5 nm). **e** Top (upper panel) and side (lower panel) views of the DFT equilibrium geometry of **8**. The out-of-plane buckling of the buckybowl to assume the bowl-opening-down conformation is evident. **f** STS spectra on **8** showing differential conductance peaks at −1.15 V and +2.70 V corresponding to the highest occupied molecular orbital (HOMO) and the lowest unoccupied molecular orbital (LUMO). A reference spectrum on Au(111) is shown in gray and serves as marker for the tip quality. The spectra are vertically shifted for clarity. Inset: STM topography image of **8** with overlaid DFT-optimized molecular model. The red and blue circles mark the positions at which the corresponding STS spectra were acquired. **g**, **i** Experimental d*I*/d*V* maps at the energies of HOMO and LUMO; and **h**, **j** corresponding DFT-simulated d*I*/d*V* maps at a tip-sample distance of 5 Å. (Open feedback parameters for the STS spectra: *V* = −1.6 V, *I* = 50 pA, *V*_rms_ = 20 mV, *f* *=* 860 Hz. Open feedback parameters for the experimental d*I*/d*V* maps: **d** *V* = −1.15 V, *I* = 40 pA, *V*_rms_ = 20 mV, *f* *=* 860 Hz; **i** *V* = 2.70 V, *I* = 40 pA, *V*_rms_ = 20 mV, *f* *=* 860 Hz.) All scale bars: 0.5 nm

After further annealing the surface to 320 °C, large-scale STM images show appearance of a new species (31% of 181 molecules, referred to as **8**) with apparent hexagonal shape and no prominent lobes (Fig. 4a, highlighted with the white circles). Furthermore, concomitant with the appearance of **8** is a decrease in the amount of half-closed species **7b** on the surface (42% of 181 molecules). Figure 4b shows a high-resolution STM image of **8** acquired with a CO tip, where the presence of six bays at the periphery of molecules clearly hints toward complete ring closure. Finally, the UHR-STM image of **8** (Fig. 4c) unambiguously proves the structure of the intended nitrogen-embedded buckybowl. We note that current state-of-the-art scanning probe techniques used for chemical structure visualization, i.e., noncontact atomic force microscopy and UHR-STM, show no appreciable contrast between carbon and nitrogen atoms evident through previous structural studies employing these techniques on nitrogen-doped graphene nanostructures^37,38^. This is reflected in the UHR-STM image of **8** where the presence of nitrogen atoms is not apparent. However, any ambiguity regarding the loss of nitrogen atoms from the framework of **8** is alleviated by the fact that Au(111) is a relatively mild substrate for catalyzing on-surface reactions, and previous works on synthesis of nitrogen-doped graphene nanostructures on Au(111), with temperatures similar to or higher than those reported in our work, have shown no loss of heteroatoms from the corresponding organic frameworks^39,40^. DFT calculations suggest that the molecule adopts a dome-shaped bowl-opening-down conformation on the surface (Fig. 4e, f; also see Supplementary Figs. 25 and 26 for bowl-inversion energetics of **8** in the gas-phase and on Au(111), respectively), which is further confirmed by the excellent agreement between the experimental and simulated STM images (c.f. Fig. 4b, d). The computed bowl depth of the buckybowl on Au(111) amounts to 1.2 Å (bowl depth in the gas-phase amounts to 1.4 Å, see Supplementary Fig. 25). In this respect, the UHR-STM technique is evidently advantageous to gain chemical structure information on geometrically corrugated systems, such as **8**. Surface-adsorbed buckybowls are known to show a preference for a bowl-opening-up conformation as a consequence of maximizing the van der Waals interaction between the molecule and the underlying surface. So far, bowl-inversion of surface-adsorbed buckybowls to adopt a bowl-opening-down conformation has been reported to occur either as a result of interplay between intermolecular and molecule–substrate interaction in an ensemble of buckybowls^41^, or locally due to perturbation with the STM tip^42^. Similar bidirectional conformational switching has also been observed in supramolecular assemblies of surface-supported porphyrin derivatives^43^. Interestingly, coronene molecules were also shown to adopt a dome-shaped conformation after complete dehydrogenation on a Ir(111) surface^44^. However, to the best of our knowledge, we show here the unprecedented case of isolated surface-adsorbed buckybowls with a preferential bowl-opening-down conformation (see Supplementary Fig. 27 for a discussion pertaining to the origin of the bowl-opening-down conformation of **8** on the surface). This class of bowl-opening-down buckybowls may provide a template to intercalate atomic species underneath such structures, allowing for atomic-scale tuning of their chemical or electronic properties, and potential application in emerging fields, such as spintronics^45^. It is worth to mention that coexistent with the majority bowl-opening-down buckybowls, a minor species (referred to as **8′**) is also present on the surface. Our analysis suggests that these minority species are actually the bowl-opening-up form of **8** (see Supplementary Fig. 22 and accompanying text). Finally, after annealing the sample to 350 °C (see Supplementary Fig. 21), most of the molecules undergo intermolecular coupling, with **8** as the predominant species on the surface (60% of 75 molecules). At this juncture, a comment regarding the markedly different efficiencies of **6a** and **6b** in obtaining **8** is imperative. From a DFT-based structural analysis, **6a** and **6b** exhibit similar distances of ~3 Å between neighboring uncyclized carbon atoms (Figs. 2d and 3d). Compared to the respective precursor molecules, **7a** and **7b** feature an increase in the distance between corresponding carbon atoms which remain uncyclized, it being 3.3 Å for **7a** and 3.1 Å for **7b** (Figs. 2i and 3i). The 0.2 Å larger C–C distance in **7a** compared to **7b** may explain the unfeasibility of **7a** to yield **8** at our reaction conditions, as a larger C–C distance would imply the need for higher temperatures to attempt ring closure, and other competing reaction pathways may dominate at higher temperatures.

We also performed STS measurements on **8** to probe its detailed electronic structure. The right panel of Fig. 4 shows the result of STS measurements. Voltage-dependent differential conductance spectra (d*I*/d*V* vs. *V*) show strong peaks in the density of states at −1.15 V and +2.70 V (Fig. 4f and Supplementary Fig. 24), which are assigned to the positive and negative ion resonances deriving from the highest occupied and lowest unoccupied molecular orbitals (HOMO and LUMO) of **8**, respectively. Figure 4g, i show corresponding constant-height d*I*/d*V* maps at the energetic positions of HOMO and LUMO. The conductance feature associated with the HOMO appears elliptical in shape and has maximum intensity at the center of the molecule, with its long axis oriented approximately along the heptagonal rings. The LUMO appears as two bright lobes of high intensity around the center, with additional features of weaker intensity at the bay positions of the molecule. Interpretation of these d*I*/d*V* maps is facilitated via DFT-calculated LDOS maps of HOMO and LUMO for **8** on Au(111) surface (Fig. 4h, j), evaluated at a height of 5 Å above the molecule to account for a realistic comparison to experimental conditions. Indeed, the calculated LDOS maps show excellent agreement with the experimental d*I*/d*V* maps, reinforcing the chemical identity of the molecule and allowing us to conclude that features present in the experimental d*I*/d*V* maps in Fig. 4g, i can indeed be correlated with the spatial distribution of frontier orbital wavefunctions. Regarding the appearance of d*I*/d*V* maps, it must be mentioned that the experimentally observed limited spatial extension of frontier orbitals in or around the center of the molecule should not be interpreted as a localized conductance feature. We rather attribute this feature to a combined effect of the geometrical corrugation of the buckybowl (as revealed by DFT calculations in Fig. 4e), where the STM tip that scans at a constant height above the Au(111) surface will be significantly closer to the central part of the buckybowl than the peripheral part; and the joint effect of the tip-sample distance and the parity of sample orbital wavefunctions, as has been shown previously in STS investigations on graphene nanoribbons^46^. These factors, taken together, may explain our observation of a weaker d*I*/d*V* signal at the periphery and comparatively stronger signal in or around the central part of the buckybowl. In fact, the DFT-calculated frontier orbital wavefunctions of **8** are found to be delocalized over the entire molecule (see Supplementary Fig. 23).

Topologically, the core of **8** consists of a pair of joined pentagonal rings in between a pair of heptagonal rings, identified as the ISTW defect in graphene. The ISTW defect differs in the topology of the non-hexagonal rings from the more prevalent STW defect, which consists of a pair of joined heptagonal rings in between a pair of pentagonal rings^47–50^. So far, the ISTW defect has been experimentally demonstrated to occur on single layer graphene under a transmission electron microscope (TEM) either via high-energy electron irradiation, causing removal of atoms and subsequent reconstruction of the carbon lattice around vacancies^51^, or low-energy landing of carbon atom(s) and their subsequent incorporation as dimers in the sp^2^ framework^52^. While promising, the short lifetime of such defects under the electron beam of a TEM and the random nature of generation with no control over spatial location precludes their application in nanoscale defect engineering of graphene for device applications. We demonstrate here the first controllable synthesis of the ISTW topology embedded in a buckybowl. The possibility of tailoring the chemical and electronic properties of graphene^53,54^ and carbon nanotubes^55,56^ by such topological defects has been theoretically discussed. We expect that our combined solution/surface synthetic approach will provide future avenues toward fabrication of such exotic non-hexagonal ring topologies with atomic precision, with the possibility of heteroatom substitution to further tailor their properties; and provide precise control over their spatial location in graphene flakes and extended graphenic structures. It is interesting to note that the dome-shaped conformation of the synthesized buckybowl agrees with the theoretical prediction of the out-of-plane buckling of a graphene sheet due to the strain imposed by the ISTW topology, creating nanoscale “blisters”^54^. As an outlook, we propose to incorporate the ISTW topology in two-dimensional graphenic sheets via on-surface Ullmann-like coupling of suitably designed precursor molecules to create designer graphene supracrystals^57^. Finally, a short discourse regarding the merits and shortcomings of on-surface synthetic methodology is appropriate. It is evident that on-surface synthesis provides for a powerful synthetic toolbox to fabricate complex nanostructures, which are otherwise difficult or impossible to achieve in solution phase. Furthermore, this approach allows for a detailed structural and electronic characterization at the atomic scale via scanning probe microscopies. However, submonolayer coverage of products obtained on the surface and the difficulty of post-synthetic transfer of products to insulating substrates or organic solvents poses challenges toward device fabrication and employment of bulk characterization techniques. Thus, while on-surface synthesis opens new avenues in the fabrication and characterization of novel materials with great potential, its limited scalability currently stands as a challenge toward device applications. Nevertheless, recent progress in post-synthetic transfer techniques for surface to device translation holds great promise in this regard^58^.

## Discussion

We developed a concise synthetic pathway to the first buckybowl containing the PP core. Starting from the parent TAPP, the nitrogen-embedded buckybowl was obtained via successive ring-closing steps through in-solution oxidative aromatic coupling and on-surface cyclodehydrogenation on Au(111). The buckybowl represents the first discrete molecule possessing the ISTW topology, leading to the unprecedented case of isolated surface-adsorbed buckybowls with a preferential bowl-opening-down conformation. The electron-rich fully fused nitrogen-embedded buckybowl assuming curved bowl-like architecture may serve as an ideal candidate for a host-guest study and may play a role as a foundation for further π-extension. This work shows the potential of on-surface synthesis toward the formation of unprecedented π-extended aza-analogs of graphene and atomically precise incorporation of challenging non-hexagonal ring topologies as a route to tailor the electronic and chemical properties of graphenic structures and provide a ground for new functionalities.

## Methods

### Synthesis

The synthesis of compounds **4a**, **4b**, **5a**, **5b**, **6a**, and **6b** is described in detail in the Supplementary Information (see Supplementary Methods). For NMR spectra of the compounds reported in this article, see Supplementary Figs. 3-18. For X-ray crystallographic data of compounds **4b** and **6c**, see Supplementary Figs. 1 and 2.

### Sample preparation and STM measurements

STM experiments were performed with a commercial low-temperature STM (*T*~5 K) from ScientaOmicron operating at base pressure below 1 × 10^−10^ mbar. Au(111), Ag(111), and Cu(111) single crystal surfaces were prepared by iterated cycles of sputtering with Ar^+^ ions (*p* = 6 × 10^−6^ mbar) and annealing to 750 K for 20 min at pressures below 5 × 10^−10^ mbar. STM was used to check the quality of the surfaces before deposition of molecules. Precursor molecules **6a** and **6b** were sublimed at 583 and 603 K, respectively, on the single crystal surfaces held at room temperature. Owing to the large distance between the evaporator and the substrate, only ~2% of the sublimated molecules reach the substrate. All STM images were acquired in constant-current mode, unless otherwise noted. Indicated tunneling bias voltages are given with respect to the sample. Unless otherwise mentioned, gold-coated tungsten tips were used for STM imaging and spectroscopy. Differential conductance spectra and associated maps were measured using the lock-in technique to obtain a signal proportional to d*I*/d*V* from the first harmonic of the tunneling current. UHR-STM images were acquired with a CO-terminated tip in constant-height mode, at biases close to the Fermi energy, and the current signal was recorded. Open feedback parameters and subsequent approach distances (Δ*z*) are given in respective figure descriptions. CO-terminated tips were obtained by picking up individual CO molecules from NaCl islands deposited after completion of on-surface reactions. NaCl islands facilitate the identification and pickup of individual CO molecules. All STM and d*I*/d*V* images were processed and analyzed with WSxM software^59^.

### Calculation methods

To obtain the equilibrium geometries of the molecules adsorbed on the Au(111) substrate and to compute corresponding STM images, we used the CP2K code^60,61^ implementing DFT within a mixed Gaussian plane waves approach^62^. The surface/adsorbate systems were modeled within the repeated slab scheme^63^, i.e., a simulation cell contained four atomic layers of Au along the [111] direction, a layer of hydrogen atoms to passivate one side of the slab (in order to suppress one of the two Au(111) surface states) and 40 Å of vacuum to decouple the system from its periodic replicas in the direction perpendicular to the surface. The electronic states were expanded with a TZV2P Gaussian basis set^64^ for C and H species, and a DZVP basis set for N and Au species. A cutoff of 600 Ry was used for the plane wave basis set. Norm-conserving Goedecker–Teter–Hutter^65^ pseudopotentials were used to represent the frozen core electrons of the atoms. We used the PBE parameterization for the generalized gradient approximation of the exchange correlation functional^66^. To account for van der Waals interactions we used the scheme proposed by Grimme et al.^67^. We considered supercells of 41.64 × 41.21 Å corresponding to 224 surface units. To obtain the equilibrium geometries we kept the atomic positions of the bottom two layers of the slab fixed to the ideal bulk positions, all other atoms were relaxed till forces were lower than 0.005 eV/Å.

To obtain simulated STM images^68^, within the Tersoff–Hamann approximation^69^, we extrapolated the electronic orbitals to the vacuum region in order to correct the wrong decay of the charge density in vacuum due to the localized basis set. The inversion energy barrier for the molecule in the gas-phase was obtained by means of the nudged elastic band method^70^, employing nine replicas.

### Data availability

X-ray crystallographic data for **4b** and **6c** can be obtained free of charge from the Cambridge Crystallographic Data Centre (CCDC) with the CCDC numbers 1589417 (**4b**) and 1589418 (**6c**) via its website (https://www.ccdc.cam.ac.uk/structures/). All other data that support the findings of this study are available from the corresponding authors upon reasonable request.

## Electronic supplementary material

Supplementary Information

Descriptions of Additional Supplementary Files

Supplementary Data 1

Supplementary Data 2

Supplementary Data 3

Supplementary Data 4

## References

[1] Tsefrikas VM, Scott LT (2006). Geodesic polyarenes by flash vacuum pyrolysis. Chem. Rev..

[2] Rabideau PW, Sygula A (1996). Buckybowls: polynuclear aromatic hydrocarbons related to the buckminsterfullerene surface. Acc. Chem. Res..

[3] Bock H (2014). Helicenes from diarylmaleimides. Org. Lett..

[4] Casas-Solvas JM, Howgego JM, Davis AP (2014). Synthesis of substituted pyrenes by indirect methods. Org. Biomol. Chem..

[5] Ball M (2015). Contorted polycyclic aromatics. Acc. Chem. Res..

[6] Narita A, Wang XY, Feng X, Müllen K (2015). New advances in nanographene chemistry. Chem. Soc. Rev..

[7] Scott LT, Hashemi MM, Meyer DT, Warren HB (1991). Corannulene. A convenient new synthesis. J. Am. Chem. Soc..

[8] Seiders TJ, Baldridge KK, Siegel JS (1996). Synthesis and characterization of the first corannulene cyclophane. J. Am. Chem. Soc..

[9] Seiders TJ, Elliott EL, Grube GH, Siegel JS (1999). Synthesis of corannulene and alkyl derivatives of corannulene. J. Am. Chem. Soc..

[10] Povie G, Segawa Y, Nishihara T, Miyauchi Y, Itami K (2017). Synthesis of a carbon nanobelt. Science.

[11] Steinberg BD (2009). Aromatic π-systems more curved than C60. The complete family of all indenocorannulenes synthesized by iterative microwave-assisted intramolecular arylations. J. Am. Chem. Soc..

[12] Jackson EA, Steinberg BD, Bancu M, Wakamiya A, Scott LT (2007). Pentaindenocorannulene and tetraindenocorannulene: new aromatic hydrocarbon π systems with curvatures surpassing that of C60. J. Am. Chem. Soc..

[13] Kawasumi K, Zhang Q, Segawa Y, Scott LT, Itami K (2013). A grossly warped nanographene and the consequences of multiple odd-membered-ring defects. Nat. Chem..

[14] Fujikawa T, Segawa Y, Itami K (2016). Synthesis and structural features of quadruple helicenes: highly distorted π systems enabled by accumulation of helical repulsions. J. Am. Chem. Soc..

[15] Wang X (2014). Heteroatom-doped graphene materials: syntheses, properties and applications. Chem. Soc. Rev..

[16] Kong XK, Chen CL, Chen QW (2014). Doped graphene for metal-free catalysis. Chem. Soc. Rev..

[17] Wang H, Maiyalagan T, Wang X (2012). Review on recent progress in nitrogen-doped graphene: synthesis, characterization, and its potential applications. ACS Catal..

[18] Tzirakis MD, Orfanopoulos M (2013). Radical reactions of fullerenes: from synthetic organic chemistry to materials science and biology. Chem. Rev..

[19] Ito S, Tokimaru Y, Nozaki K (2015). Benzene-fused azacorannulene bearing an internal nitrogen atom. Angew. Chem. Int. Ed..

[20] Yokoi H (2015). Nitrogen-embedded buckybowl and its assembly with C60. Nat. Commun..

[21] Janiga A, Glodkowska-Mrowka E, Stoklosa T, Gryko DT (2013). Synthesis and optical properties of tetraaryl-1,4-dihydropyrrolo[3,2-*b*]pyrroles. Asian J. Org. Chem..

[22] Krzeszewski M, Thorsted B, Brewer J, Gryko DT (2014). Tetraaryl-, pentaaryl-, and hexaaryl-1,4-dihydropyrrolo[3,2-*b*]pyrroles: synthesis and optical properties. J. Org. Chem..

[23] Krzeszewski M, Gryko D, Gryko DT (2017). The tetraarylpyrrolo[3,2-*b*]pyrroles—from serendipitous discovery to promising heterocyclic optoelectronic materials. Acc. Chem. Res..

[24] Janiga A, Krzeszewski M, Gryko DT (2015). Diindolo[2,3-*b*:2′,3′-*f*]pyrrolo[3,2-*b*]pyrroles as electron-rich, ladder-type fluorophores: synthesis and optical properties. Chemistry.

[25] Krzeszewski M, Gryko DT (2015). χ-shaped bis(areno)-1,4-dihydropyrrolo[3,2-*b*]pyrroles generated by oxidative aromatic coupling. J. Org. Chem..

[26] Tasior M, Chotkowski M, Gryko DT (2015). Extension of pyrrolopyrrole π-system: approach to constructing hexacyclic nitrogen-containing aromatic systems. Org. Lett..

[27] Krzeszewski M (2016). Nonplanar butterfly-shaped π-expanded pyrrolopyrroles. Chemistry.

[28] Krzeszewski M (2016). The role of steric hindrance in the intramolecular oxidative aromatic coupling of pyrrolo[3,2-*b*]pyrroles. Chem. Commun..

[29] Gourdon A (2016). On-Surface Synthesis.

[30] Cai J (2010). Atomically precise bottom-up fabrication of graphene nanoribbons. Nature.

[31] Sanchez-Valencia JR (2014). Controlled synthesis of single-chirality carbon nanotubes. Nature.

[32] Krüger J (2017). Decacene: on-surface generation. Angew. Chem. Int. Ed..

[33] Rogers C (2015). Closing the nanographene gap: surface-assisted synthesis of peripentacene from 6,6′-bipentacene precursors. Angew. Chem. Int. Ed..

[34] Hieulle J (2018). On-surface route for producing planar nanographenes with azulene moieties. Nano. Lett..

[35] Temirov R, Soubatch S, Neucheva O, Lassise AC, Tautz FS (2008). A novel method achieving ultra-high geometrical resolution in scanning tunnelling microscopy. New. J. Phys..

[36] Kichin G, Weiss C, Wagner C, Tautz FS, Temirov R (2011). Single molecule and single atom sensors for atomic resolution imaging of chemically complex surfaces. J. Am. Chem. Soc..

[37] Carbonell-Sanromà E (2017). Doping of graphene nanoribbons via functional group edge modification. ACS Nano.

[38] Durr RA (2018). Orbitally matched edge-doping in graphene nanoribbons. J. Am. Chem. Soc..

[39] Zhang Y (2014). Direct visualization of atomically precise nitrogen-doped graphene nanoribbons. Appl. Phys. Lett..

[40] Cai J (2014). Graphene nanoribbon heterojunctions. Nat. Nanotechnol..

[41] Jaafar R (2014). Bowl inversion of surface-adsorbed sumanene. J. Am. Chem. Soc..

[42] Fujii S, Ziatdinov M, Higashibayashi S, Sakurai H, Kiguchi M (2016). Bowl inversion and electronic switching of buckybowls on gold. J. Am. Chem. Soc..

[43] Ditze S (2014). On the energetics of conformational switching of molecules at and close to room temperature. J. Am. Chem. Soc..

[44] Curcio D (2016). Molecular lifting, twisting, and curling during metal-assisted polycyclic hydrocarbon dehydrogenation. J. Am. Chem. Soc..

[45] Sicot M (2012). Size-selected epitaxial nanoislands underneath graphene Moiré on Rh(111). ACS Nano.

[46] Söde H (2015). Electronic band dispersion of graphene nanoribbons via Fourier-transformed scanning tunneling spectroscopy. Phys. Rev. B.

[47] Thrower PA, Mayer RM (1978). Point defects and self-diffusion in graphite. Phys. Status Solidi Appl. Res..

[48] Stone AJ, Wales DJ (1986). Theoretical studies of icosahedral C60 and some related species. Chem. Phys. Lett..

[49] Meyer JC (2008). Direct imaging of lattice atoms and topological defects in graphene membranes. Nano. Lett..

[50] Berthe M (2007). Reversible defect engineering of single-walled carbon nanotubes using scanning tunneling microscopy. Nano. Lett..

[51] Robertson AW, He K, Kirkland AI, Warner JH (2014). Inflating graphene with atomic scale blisters. Nano. Lett..

[52] Lehtinen O, Vats N, Algara-Siller G, Knyrim P, Kaiser U (2015). Implantation and atomic-scale investigation of self-interstitials in graphene. Nano. Lett..

[53] Lusk MT, Carr LD (2008). Nanoengineering defect structures on graphene. Phys. Rev. Lett..

[54] Lusk MT, Wu DT, Carr LD (2010). Graphene nanoengineering and the inverse Stone-Thrower-Wales defect. Phys. Rev. B.

[55] Orlikowski D, Buongiorno Nardelli M, Bernholc J, Roland C (1999). Ad-dimers on strained carbon nanotubes: a new route for quantum dot formation?. Phys. Rev. Lett..

[56] Sternberg M (2006). Carbon Ad-dimer defects in carbon nanotubes. Phys. Rev. Lett..

[57] Lusk MT, Carr LD (2009). Creation of graphene allotropes using patterned defects. Carbon.

[58] Llinas JP (2017). Short-channel field-effect transistors with 9-atom and 13-atom wide graphene nanoribbons. Nat. Commun..

[59] Horcas I (2007). WSXM: a software for scanning probe microscopy and a tool for nanotechnology. Rev. Sci. Instrum..

[60] CP2K, Open Source Molecular Dynamics. Available at: http://www.cp2k.org (The CP2K developers group, 2013).

[61] Hutter J, Iannuzzi M, Schiffmann F, VandeVondele J (2014). cp2k: atomistic simulations of condensed matter systems. Wiley Interdiscip. Rev. Comput. Mol. Sci..

[62] VandeVondele J (2005). Quickstep: fast and accurate density functional calculations using a mixed Gaussian and plane waves approach. Comput. Phys. Commun..

[63] Pickett WE (1989). Pseudopotential methods in condensed matter applications. Comput. Phys. Rep..

[64] VandeVondele J, Hutter J (2007). Gaussian basis sets for accurate calculations on molecular systems in gas and condensed phases. J. Chem. Phys..

[65] Goedecker S, Teter M, Hutter J (1996). Separable dual-space Gaussian pseudopotentials. Phys. Rev. B.

[66] Perdew JP, Burke K, Ernzerhof M (1996). Generalized gradient approximation made simple. Phys. Rev. Lett..

[67] Grimme S, Antony J, Ehrlich S, Krieg H (2010). A consistent and accurate ab initio parametrization of density functional dispersion correction (DFT-D) for the 94 elements H-Pu. J. Chem. Phys..

[68] Talirz, L. Toolkit using the Atomistic Simulation Environment (ASE). Available at: https://github.com/ltalirz/asetk (2015).

[69] Tersoff JD, Hamann DR (1985). Theory of the scanning tunneling microscope. Phys. Rev. B.

[70] Henkelman G, Uberuaga BP, Jónsson H (2000). A climbing image nudged elastic band method for finding saddle points and minimum energy paths. J. Chem. Phys..

