# Supplementary material for: On-surface synthesis of a nitrogen-embedded buckybowl with inverse Stone–Thrower–Wales topology
Source: Nat Commun. 2018 Apr 30;9:1714. doi: 10.1038/s41467-018-04144-5 (PMC5928119; doi:10.1038/s41467-018-04144-5)
Supplement: Supplementary file 1 — Supplementary Information [file 41467_2018_4144_MOESM1_ESM.pdf]

On-surface Synthesis of a Nitrogen-Embedded Buckybowl with Inverse Stone-Thrower-Wales Topology

Mishra *et al*.

Supplementary Methods

1. General chemical methods

All reagents and solvents were purchased from commercial sources and were used as received unless otherwise noted. Reagent grade solvents (CH_2Cl_2, hexanes) were distilled prior to use. DMF was dried over magnesium sulfate, then distilled and stored under argon. Transformations with moisture and oxygen sensitive compounds were performed under a stream of argon. The reaction progress was monitored by means of thin layer chromatography (TLC), which was performed on aluminium foil plates, covered with Silica gel 60 F_{254} (Merck) or Aluminium oxide 60 F_{254} (neutral, Merck). Products purification was done by means of column chromatography with Kieselgel 60 (Merck) or Aluminium oxide (Fluka). Occasionally, dry column vacuum chromatography (DCVC**)** for purification of products obtained was performed using Silica gel Type D 5F. The identity and purity of prepared compounds were proved by ^{1}H NMR and ^{13}C NMR spectrometry as well as by MS-spectrometry (*via* EI-MS or ESI-MS). NMR spectra were measured on Bruker AM 500 MHz, Bruker AM 600 MHz, Varian 600 MHz, Varian 400 MHz or Varian 200 MHz instruments with TMS as internal standard. All chemical shifts are given in ppm. All melting points for crystalline products were measured with automated melting point apparatus EZ-MELT and were given without correction. The absorbance and fluorescence spectra were measured in dichloromethane on Perkin – Elmer Lambda 25 UV/VIS and Hitachi F-7000 respectively.

2. **In-solution synthesis procedures**

General procedure for the synthesis of amine 2c.

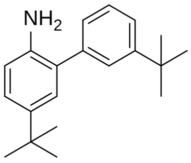

2c

2-bromo-4-*t*-butylaniline (12 mmol), 3-*t*-butylphenylboronic acid (13 mmol), K_2CO_3 (20 mmol), PPh_3 (20% mol), $Pd(OAc)_2$ (10% mol) were placed in a 100 mL Schlenk flask, which was flushed with argon prior to use. Then 40 mL of 1:1 v/v mixture of toluene and water was added, and the resulting mixture was stirred at 80 °C for 16 h. After cooling two phases were separated, water phase was extracted with ethyl acetate (3× 20 mL). Organic phases were combined and dried, solvent was evaporated and crude products were purified by means of flash column chromatography (SiO_2, Hexanes: Ethyl Acetate 6:1 to 4:1) affording 2.99 g of desired product **2c**.
2c- Brownish oil. Yield 2.99 g (89%). R_f= 0.57 (SiO_2, Ethyl Acetate/hexanes, 1:5). ^{1}H NMR (500 MHz, $CDCl_3$) δ 7.51-7.49 (m, 1H), 7.39 – 7.36 (m, 2H), 7.29-7.26 (m, 1H), 7.20 (dd, *J* = 8.3, 2.4 Hz, 1H), 7.17 (d, *J* = 2.3 Hz, 1H), 6.75 (d, *J* = 8.3 Hz, 1H), 3.59 (bs, 2H), 1.36 (s, 9H), 1.31 (s, 9H). ^{13}C NMR (126 MHz, $CDCl_3$) δ 151.6, 141.6, 140.7, 139.6, 128.4, 127.9, 127.4, 126.3, 126.2, 125.3, 124.0, 115.5, 34.8, 34.0, 31.6, 31.4. HRMS (ESI-TOF) calcd for $C_{20}H_{28}N$: 282.2222 $[M+H]^+$, found: 282.2213.

General procedure for the synthesis of Tetrarylpyrroloyrroles (4a-c)

In a 25 mL round-bottom flask equipped with a reflux condenser and magnetic stir bar, 6 mL glacial acetic acid was placed followed by the addition of aldehyde (6 mmol), 2-aminobiphenyl (6 mmol), and TsOH (0.6 mmol). The mixture was stirred at 90 °C for 30 min. After that time butane-2,3-dione (3 mmol) was slowly added via syringe and the resulting mixture was stirred at 90 °C for 3 h. The reaction mixture was then cooled to room temperature. The precipitate of the obtained dye was then filtered off and washed with cooled glacial acetic acid. Recrystallization from AcOEt and drying under vacuum afforded pure product.

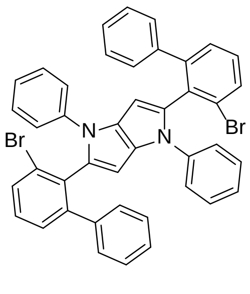

4a

4a- White solid. Yield 775 mg (36%). M.p. 250-251°C. R_f= 0.42 (SiO_2, DCM/hexanes, 1:2). ^{1}H NMR (500 MHz, $CDCl_3$) δ 7.67 (dd, *J* = 7.5, 1.8 Hz, 2H), 7.23 – 7.17 (m, 4H), 7.13 – 7.06 (m, 6H), 7.04 – 6.99 (m, 6H), 6.75 – 6.70 (m, 8H), 6.26 (s, 2H). ^{13}C NMR (126 MHz, $CDCl_3$) δ 144.9, 140.3, 139.3, 133.3, 131.8, 130.90, 129.2, 129.1, 128.5, 128.3, 127.5, 127.0, 126.6, 124.3, 123.0, 98.5. HRMS (EI) calcd for $C_{42}H_{28}N_2Br_2$: 718.0619 $[M]^+$, found: 718.0621.

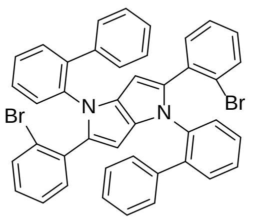

4b

4b- White solid. Yield 742 mg (33%). M.p. 228-230°C. R_f= 0.62 (SiO_2, DCM/hexanes, 1:2). Mixture of atropisomers ratio 5:1 (A:B), apparent peaks overlapping. ^{1}H NMR (500 MHz, CD_2Cl_2) δ 7.79 (dd, J = 7.8, 1.4 Hz, 10H, A), 7.56 – 7.53 (m, 2H, B), 7.49 – 7.45 (m, 10H, A), 7.41 (td, J = 7.5, 1.3 Hz, 14H, A+B), 7.34 (ddd, J = 7.3, 5.7, 1.5 Hz, 24H, A+B), 7.27 – 7.18 (m, 38H, A+B), 6.99 (dd, J = 7.4, 1.8 Hz, 2H, B), 6.97 – 6.91 (m, 16H, A+B), 6.88 (td, J = 7.4, 1.2 Hz, 10H, A), 6.79 – 6.73 (m, 20H, A), 6.57 (dd, J = 7.4, 2.0 Hz, 2H, B), 6.34 (dd, J = 7.6, 1.8 Hz, 10H, A), 6.17 (s, 10H, A), 6.04 (s, 2H, B). ^{13}C NMR (126 MHz, CD_2Cl_2) δ 139.1, 138.8, 137.1, 134.8, 134.0, 132.53, 132.49, 132.43, 132.37, 131.1, 131.0, 130.9, 129.2, 129.1, 128.3, 128.15, 128.13, 128.03, 127.98, 127.96, 127.86, 127.7, 127.5, 127.3, 127.2, 127.1, 126.3, 126.2, 123.4, 96.0, 95.3. HRMS (ESI-TOF) calcd for $C_{42}H_{28}N_2Br_2Na$: 741.0517 $[M+Na]^+$, found: 741.0498.

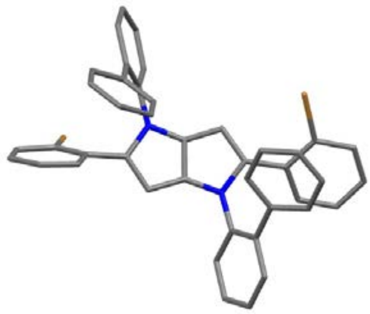

Supplementary Figure 1. Molecular structure of **4b** (hydrogen atoms omitted for clarity) (CCDC number: 1589417).

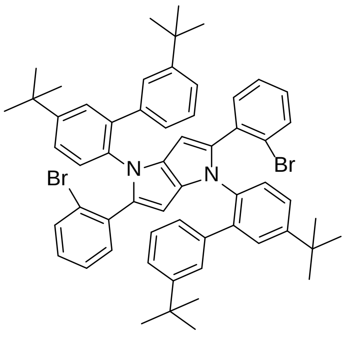

4c

4c- White solid. Yield 942 mg (33%). M.p. 289-291°C. R_f= 0.68 (SiO_2, DCM/hexanes, 1:2). Mixture of atropisomers ratio 2:9, apparent peaks overlapping. ^{1}H NMR (600 MHz, CD_2Cl_2) δ 7.66 (d, J = 8.3 Hz, 2H), 7.52 (d, J = 7.9 Hz, 9H), 7.47 (dd, J = 8.3, 2.4 Hz, 2H), 7.42 (dd, J = 8.3, 2.3 Hz, 10H), 7.36 (dd, J = 7.9, 1.4 Hz, 10H), 7.33 (d, J = 1.3 Hz, 1H), 7.32 (d, J = 2.4 Hz, 11H), 7.29 (d, J = 2.3 Hz, 2H), 7.27 (dd, J = 2.0, 1.0 Hz, 2H), 7.26 – 7.24 (m, 11H), 7.15 (t, J = 7.7 Hz, 10H), 7.09 (t, J = 7.8 Hz, 3H), 6.94 (td, J = 7.6, 1.8 Hz, 10H), 6.92 (d, J = 1.8 Hz, 2H), 6.89 (td, J = 7.5, 1.4 Hz, 12H), 6.87 – 6.85 (m, 2H), 6.82 (t, J = 1.8 Hz, 10H), 6.69 (d, J = 7.6 Hz, 10H), 6.50 (d, J = 7.9 Hz, 2H), 6.42 (dd, J = 7.6, 1.7 Hz, 11H), 6.22 (s, 2H), 6.04 (s, 9H), 1.37 (s, 18H), 1.35 (s, 81H), 1.27 (s, 18H), 1.16 (s, 81H). ^{13}C NMR (150 MHz, CD_2Cl_2) δ 150.9, 150.8, 139.5, 139.0, 135.0, 134.9, 134.6, 133.1, 132.7, 129.0, 128.4, 128.3, 128.1, 126.5, 125.9, 125.8, 125.1, 124.4, 95.6, 54.2,

54.0, 53.8, 53.7, 53.5, 34.9, 34.8, 31.6, 31.5. HRMS (EI) calcd for $C_{58}H_{60}N_2Br_2$: 942.3123 $[M]^+$, found: 942.3120.

General procedure for the synthesis of π-expanded Tetraarylpyrrolopyrroles (5a-c)

To a 20mL sealed tube equipped with magnetic stir bar (flushed with argon prior to use), 8 mL of dry toluene and 0.5 mmol of adequate TAPP were placed. To a dissolved substrate 11 mg (0.05 mmol) of $Pd(OAc)_2$, 391 mg (1.2 mmol) of Cs_2CO_3 and 30 mg (0.11 mmol) of PPh_3 were added. Reaction was conducted at 120 °C for 3 hours. Then after cooling 8 mL of water was added and resulting mixture was stirred for another 15 min. Two phases were separated, water phase was extracted with ethyl acetate (3 × 10 mL). Organic phases were combined and dried, solvent was evaporated and crude product was washed with copious amount of diethyl ether.

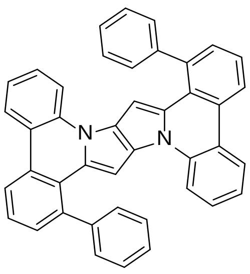

5a

5a- Yellow solid. Yield 223 mg (80%). M.p. > 400°C. R_f= 0.40 (SiO_2, DCM/hexanes, 1:2). ^1H NMR (500 MHz, *o*-DCB-d_4) δ 8.04 (dd, J = 14.2, 8.0 Hz, 4H), 7.48 (dd, J = 7.2, 1.1 Hz, 2H), 7.46 – 7.41 (m, 4H), 7.36 (dq, J = 6.8, 1.2 Hz, 4H), 7.34 – 7.31 (m, 2H), 7.29 – 7.25 (m, 2H), 7.23 – 7.18 (m, 2H), 7.18 – 7.13 (m, 2H), 7.12-7.08 (m, 2H), 5.74 (s, 2H). ^{13}C NMR (126 MHz, *o*-DCB-d_4) δ 155.6, 143.9, 138.6, 134.5, 131.5, 130.7, 128.8, 128.2, 125.7, 125.4, 124.8, 123.5, 122.4, 121.9, 121.1, 115.0, 94.1. HRMS (EI) calcd for $C_{42}H_{26}N_2$: 558.2096 $[M]^+$, found: 558.2094.

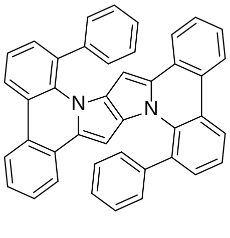

5b

5b- Yellow solid. Yield 223 mg (80%). M.p. > 400°C. R_f= 0.50 (SiO_2, DCM/hexanes, 1:2). 1H NMR (500 MHz, *o*-DCB-d_4, 80 °C) δ 7.98 (d, J = 7.9 Hz, 2H), 7.89 (d, J = 7.9 Hz, 2H), 7.40 (d, J = 8.1 Hz, 2H), 7.32 (d, J = 7.4 Hz, 2H), 7.25 (d, J = 7.1 Hz, 4H), 7.23 – 7.17 (m, 6H), 7.15 (t, J = 7.7 Hz, 2H), 7.12-7.08 (m, 4H), 5.56 (s, 2H). ^{13}C NMR (126 MHz, *o*-DCB-d_4, 25 °C) δ 141.7, 131. 7, 128.8, 128.5, 128.1, 125.4, 125.2, 124.9, 123.8, 122.7, 122.5, 122.1, 121.7, 110.0, 92.3. HRMS (ESI-TOF) calcd for $C_{42}H_{26}N_2$: 558.2096 $[M]^+$, found: 558.2077.

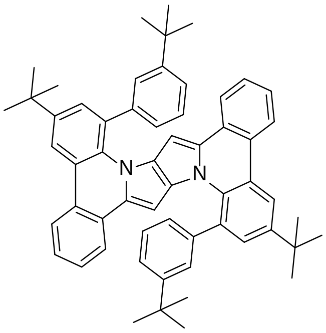

5c

5c- Yellow solid. Yield 355 mg (91%). M.p. > 400°C. R_f= 0.63 (SiO_2, DCM/hexanes, 1:2). Mixture of atropisomers ratio 2:4.4, apparent peaks overlapping. ^{1}H NMR (500 MHz, *o*-DCB-d$_4$) δ 8.19 (d, J = 2.2 Hz, 6H), 8.07 (t, J = 8.0 Hz, 6H), 7.59 (d, J = 2.1 Hz, 4H), 7.54 (s, 2H), 7.49 – 7.42 (m, 15H), 7.35 – 7.21 (m, 25H), 7.18-7.10 (m, 7H), 5.48 (s, 4H), 5.26 (s, 2H), 1.33 (s, 40H), 1.30 (s, 18H), 1.08 (s, 40H), 0.94 (s, 18H). ^{13}C NMR (125 MHz, *o*-DCB-d$_4$) δ 151.2, 150.9, 145.1, 144.8, 142.2, 141.5, 131.9, 131.7, 131.5, 130.7, 130.2, 129.36, 129.31, 128.5, 128.3, 128.0, 127.75, 127.66, 127.59, 127.3, 126.5, 125.4, 125.3, 124.0, 123.4, 122.5, 122.0, 121.9, 118.9, 118.7, 92.8, 92.0, 34.4, 34.2, 31.23, 31.18, 30.9, 30.8. HRMS (EI) calcd for $C_{58}H_{58}N_2$: 782.4600 $[M]^+$, found: 782.4612.

General procedure for the synthesis of 6a

To a 50 mL round-bottom flask, flushed with argon prior to use, and equipped with magnetic stir bar and septum, 10 mL of dry methylene chloride and 0.2 mmol of **5a** were placed. To suspended substrate, 4 mmol of iron(III) chloride dissolved in 6 mL of dry nitromethane were added via syringe. Reaction was conducted at room temperature for 30 min. Then 12 mL of water was added, and resulting mixture was stirred for another 15 min. Two phases were separated, water phase was extracted with methylene chloride (3 × 15 mL). Organic phases were combined and dried, solvent was evaporated and crude product was purified by means of flash column chromatography.

General procedure for the synthesis of 6b-c

To a 50 mL round-bottom flask, flushed with argon prior to use, and equipped with magnetic stir bar and septum, 15 mL of dry dichloroethane and 0.2 mmol of adequate π-exp TAPP **5** were placed. To dissolved substrate, 4 mmol of iron(III) chloride dissolved in 6 mL of dry nitromethane were added *via* syringe. Reaction was conducted at 80 °C for 16 hours. Then 20 mL of water was added and resulting mixture was stirred for another 15 minutes. Two phases were separated, water phase was extracted with methylene chloride (3 x 15 mL). Organic phases were combined and dried, solvent was evaporated and crude product was purified by means of flash column chromatography.

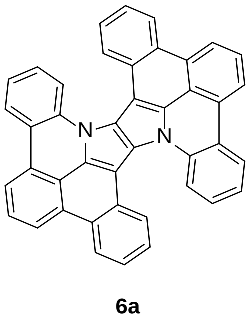

6a

6a- Yellow solid. Yield 100 mg (90%). M.p. > 360-362°C (subl.). R_f= 0.42 (SiO_2, DCM/hexanes, 1:2). NMR spectra have not been recorded due to very low solubility of the compound. HRMS (EI) calcd for $C_{42}H_{22}N_2$: 554.1783 $[M]^+$, found: 554.1788.

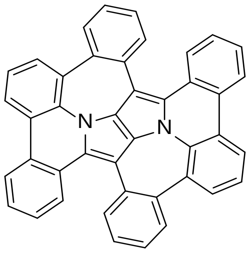

6b

6b- Orange solid. Yield 66 mg (60%). M.p. > 400°C. R_f= 0.52 (SiO_2, DCM/hexanes, 1:2). NMR spectra have not been recorded due to very low solubility of the compound. HRMS (EI) calcd for $C_{42}H_{22}N_2$: 554.1783 $[M]^+$, found: 554.1783.

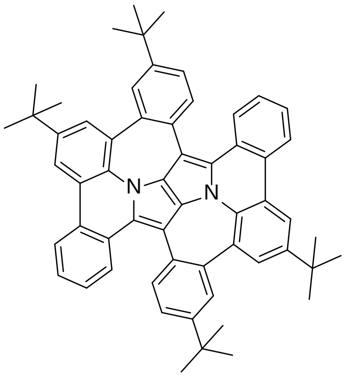

6c

6c- Orange solid. Yield 90 mg (56%). M.p. > 400°C. R_f= 0.67 (SiO_2, DCM/hexanes, 1:2). ^{1}H NMR (500 MHz, C_6D_6) δ 8.44 – 8.40 (m, 2H), 8.19 (d, J = 8.3 Hz, 2H), 7.98 (dd, J = 6.1, 3.3 Hz, 2H), 7.95 (d, J = 2.1 Hz, 2H), 7.77 (d, J = 2.1 Hz, 2H), 7.37 (d, J = 2.2 Hz, 2H), 7.18 (dd, J = 6.0, 3.2 Hz, 4H), 7.01 (dd, J = 8.3, 2.1 Hz, 2H), 1.36 (s, 18H), 1.25 (s, 18H). ^{13}C NMR (126 MHz, C_6D_6) δ 152.5, 148.5, 141.7, 138.7, 136.3, 135.8, 134.2, 134.0, 133.4, 131.7, 129.8, 129.4, 129.2, 128.1, 127.4, 127.0, 126.4, 123.8, 122.0, 110.0, 37.0, 36.9, 33.8, 33.6. HRMS (EI) calcd for $C_{58}H_{54}N_2$: 778.4287 $[M]^+$, found: 778.4305.

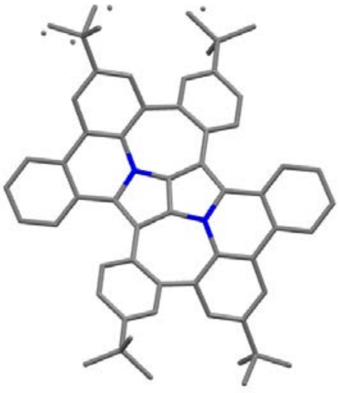

Supplementary Figure 2. Molecular structure of **6c** (hydrogen atoms omitted for clarity) (CCDC number: 1589418).

3. ^{1}H and ^{13}C NMR spectra for synthesized compounds

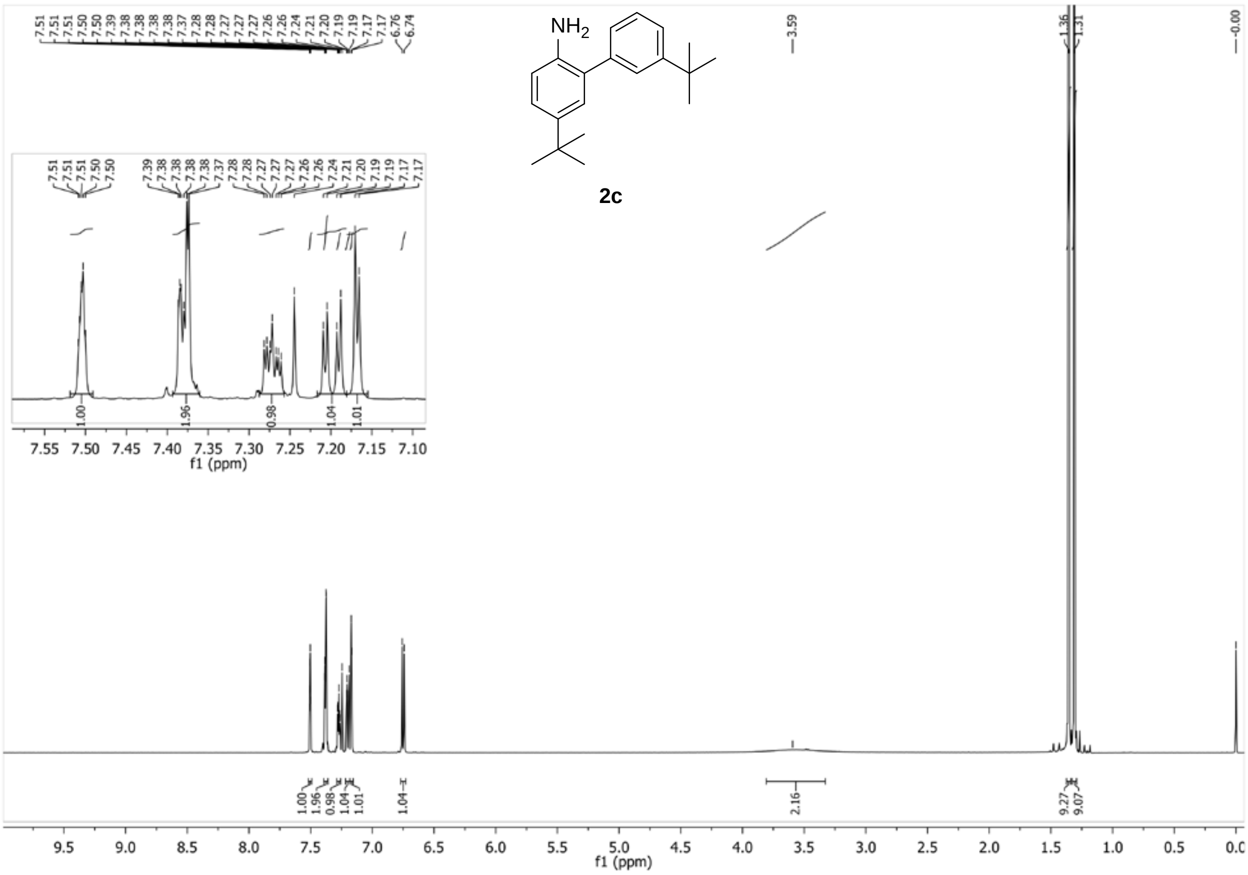

Supplementary Figure 3. ^{1}H NMR spectrum of **2c**.

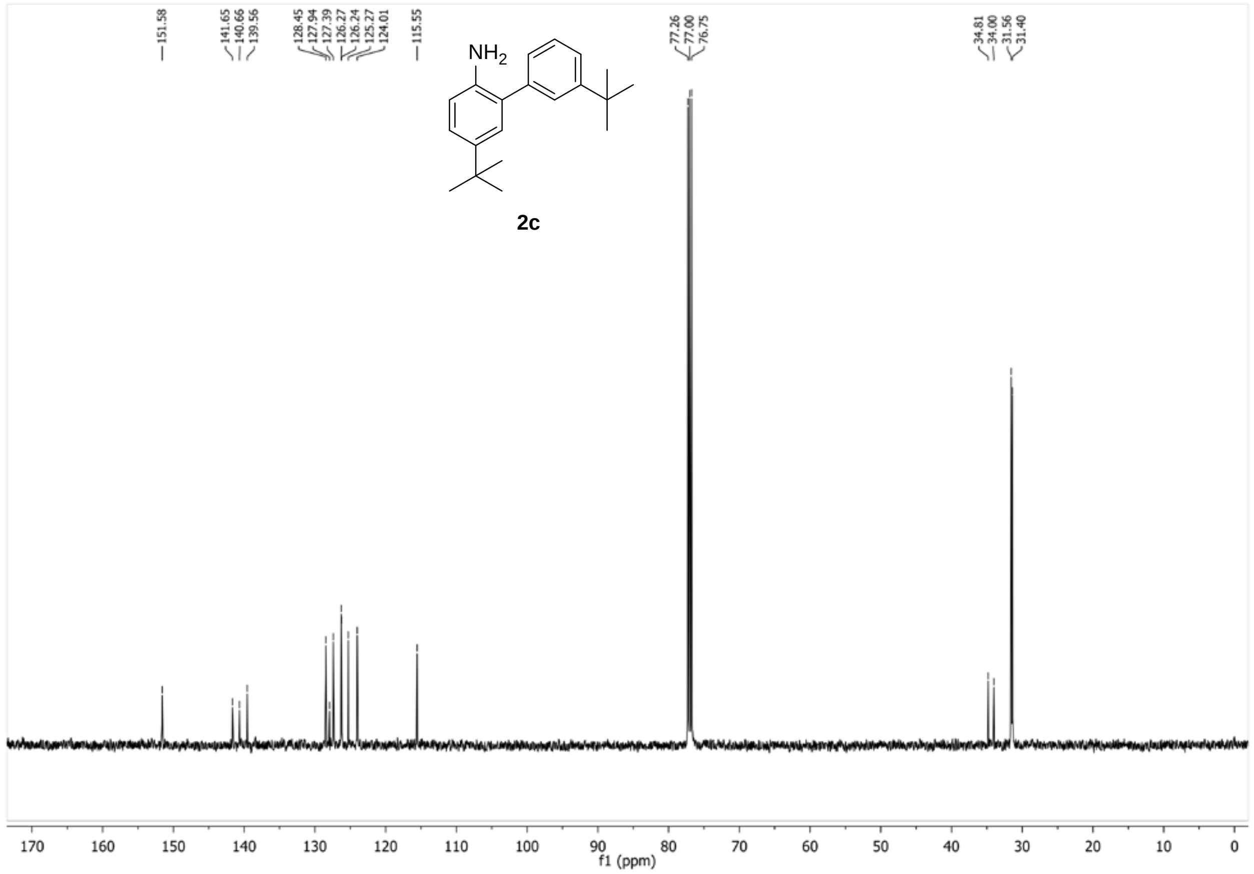

Supplementary Figure 4. ^{13}C NMR spectrum of **2c**.

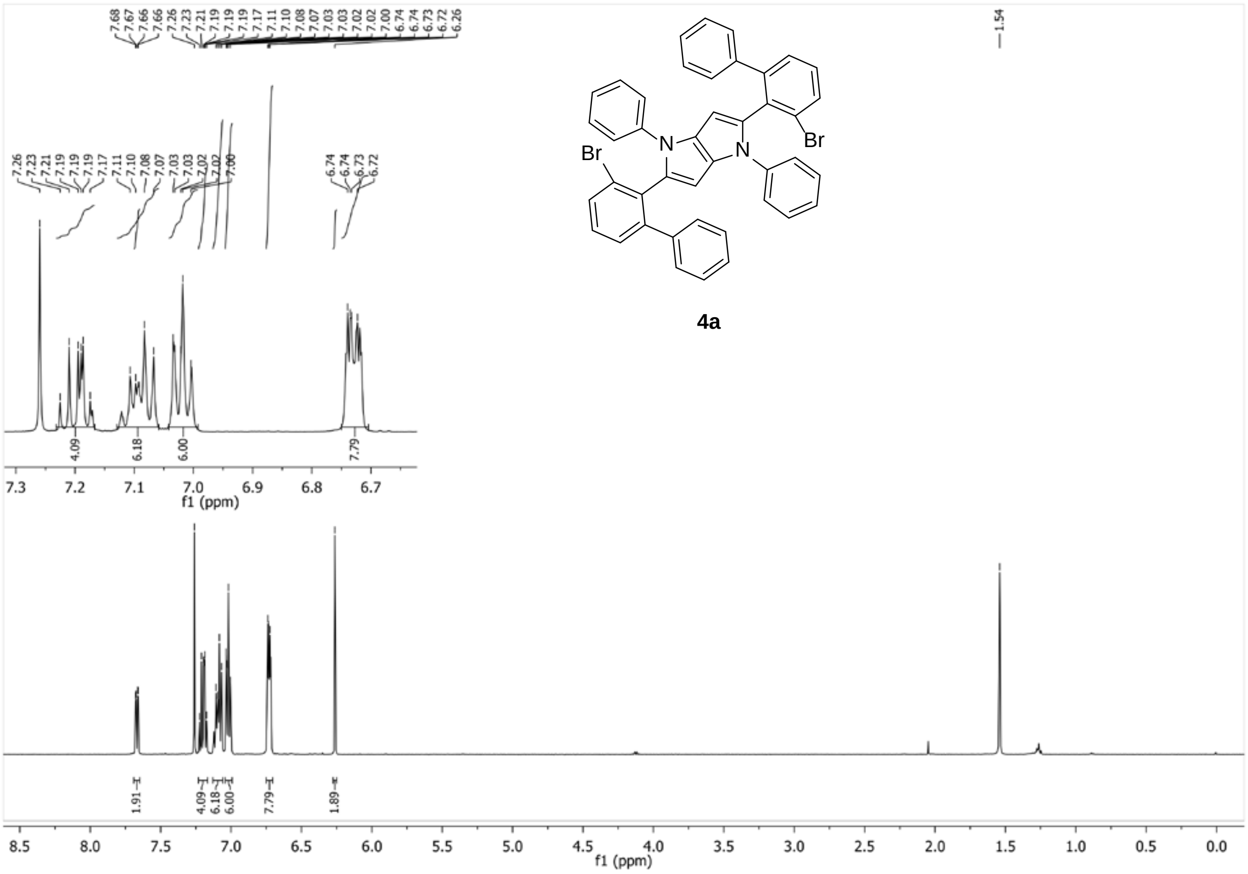

Supplementary Figure 5. ^{1}H NMR spectrum of **4a**.

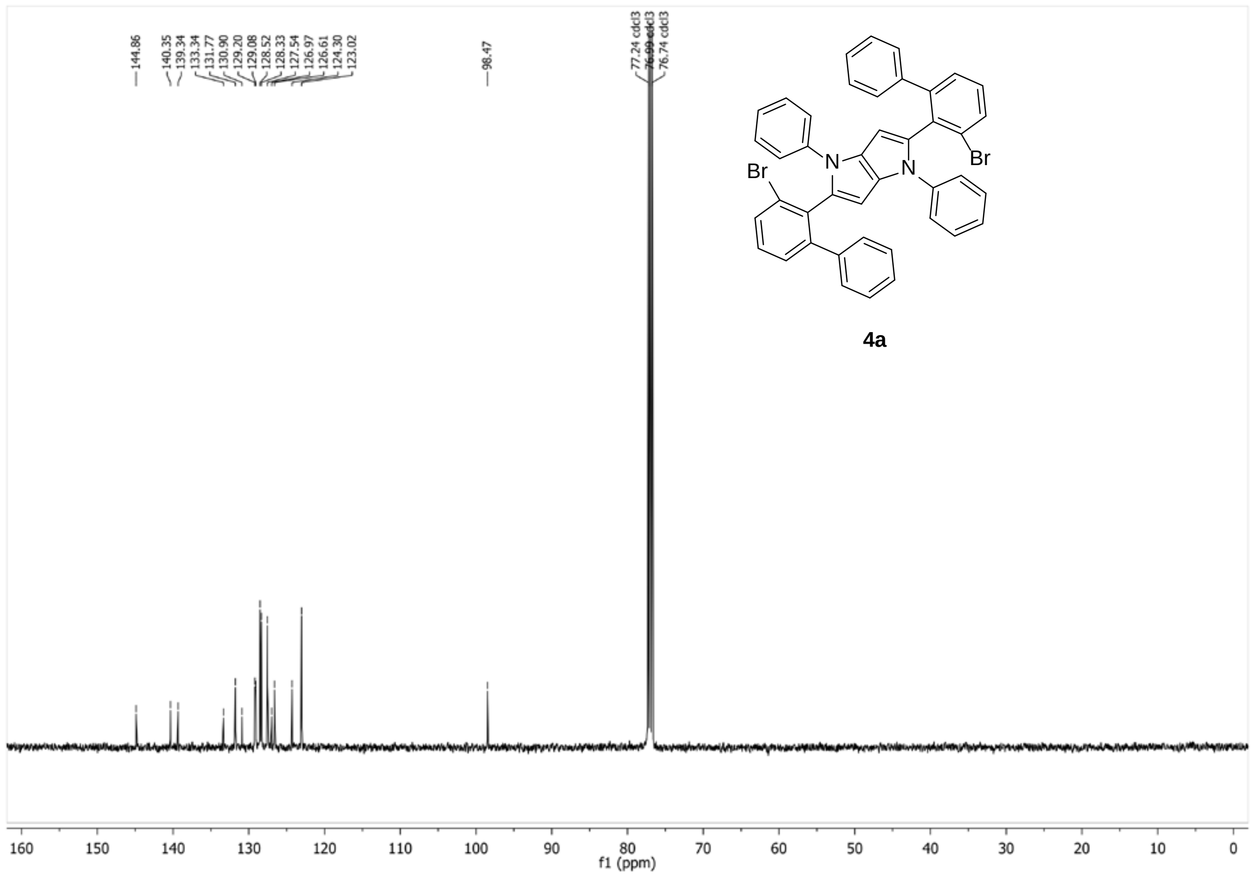

Supplementary Figure 6. ^{13}C NMR spectrum of **4a**.

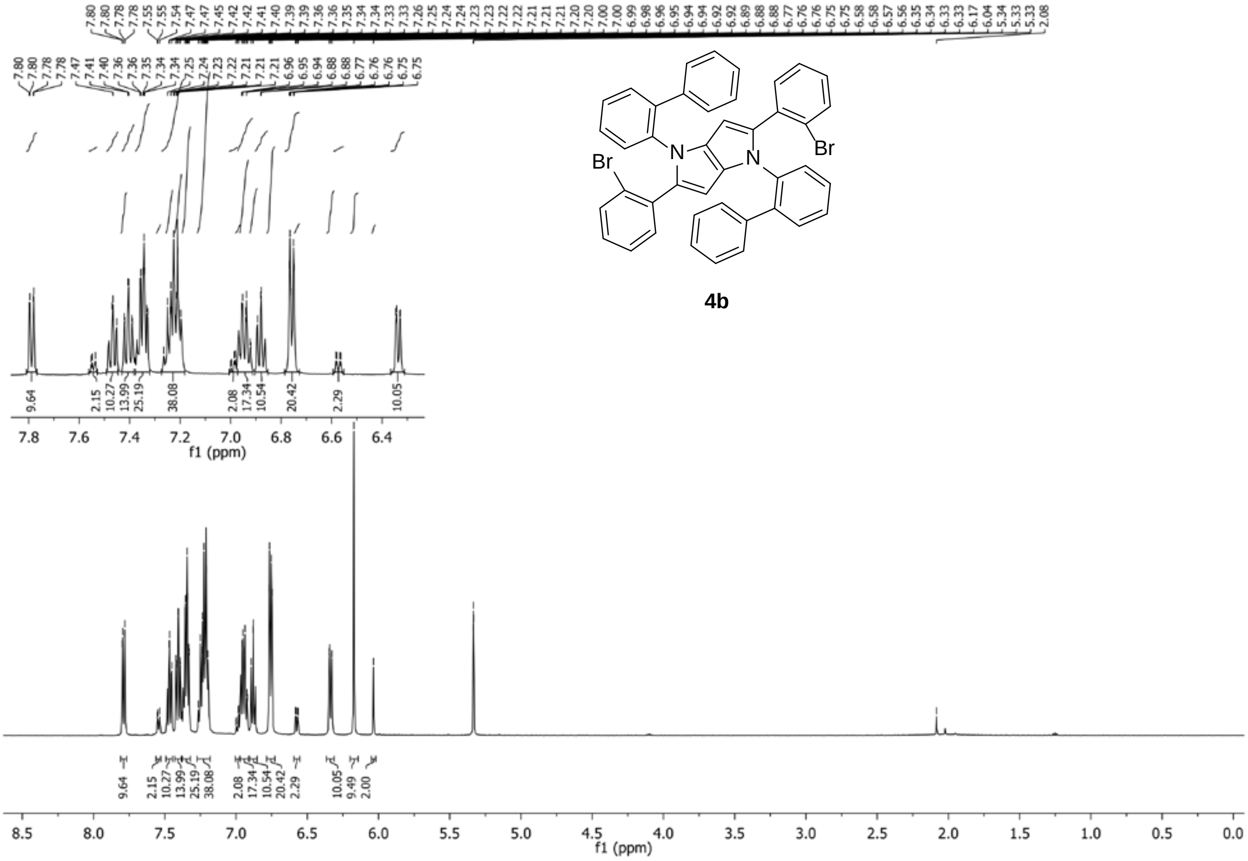

Supplementary Figure 7. ^{1}H NMR spectrum of **4b**.

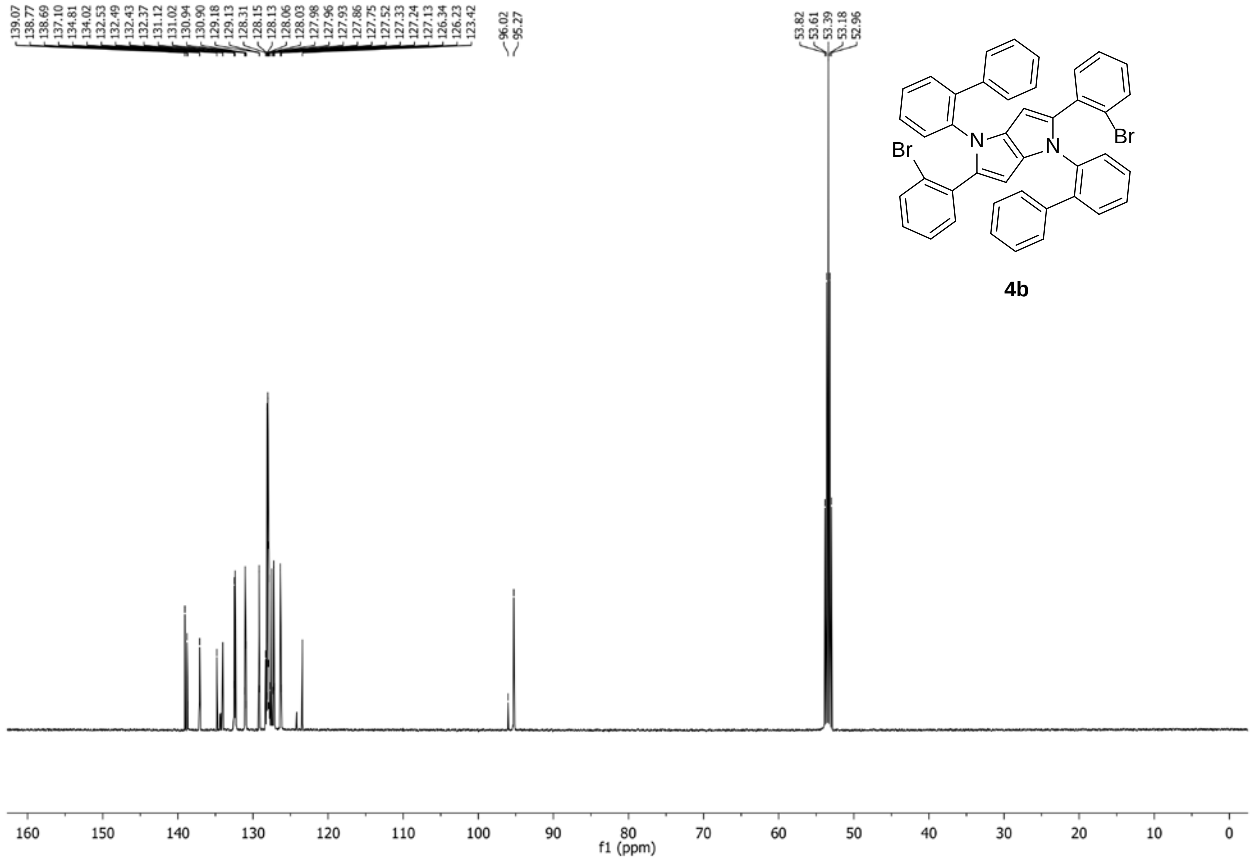

Supplementary Figure 8. ^{13}C NMR spectrum of **4b**.

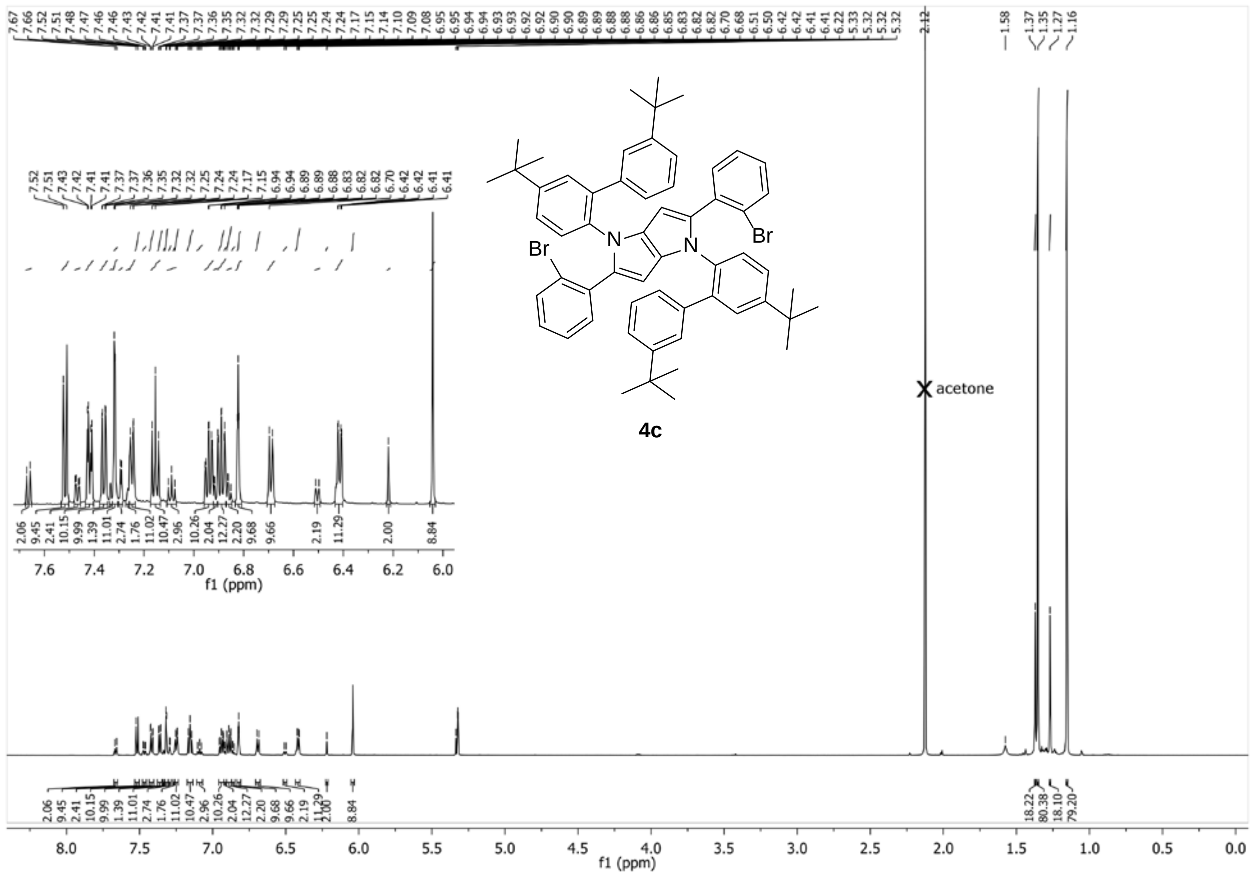

Supplementary Figure 9. ^{1}H NMR spectrum of **4c**.

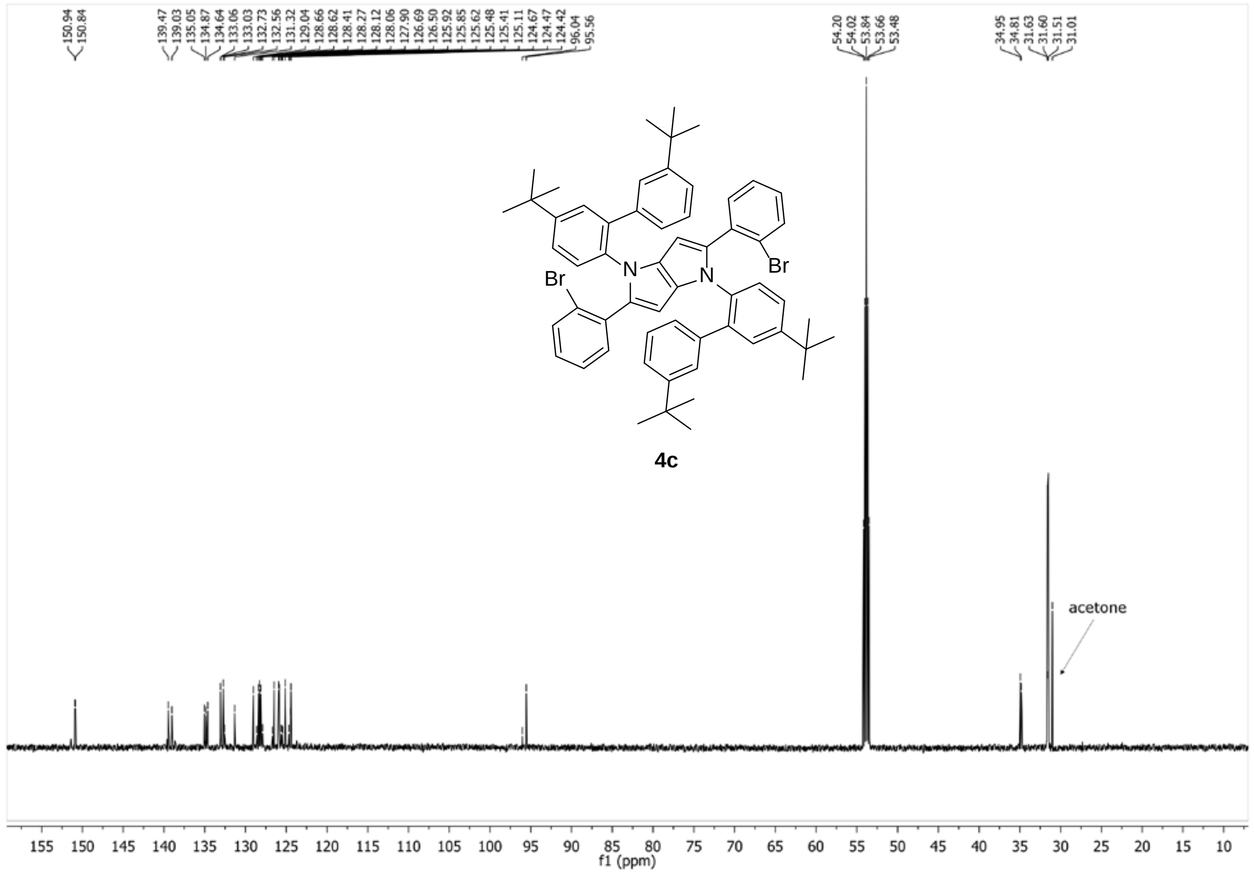

Supplementary Figure 10. ^{13}C NMR spectrum of **4c**.

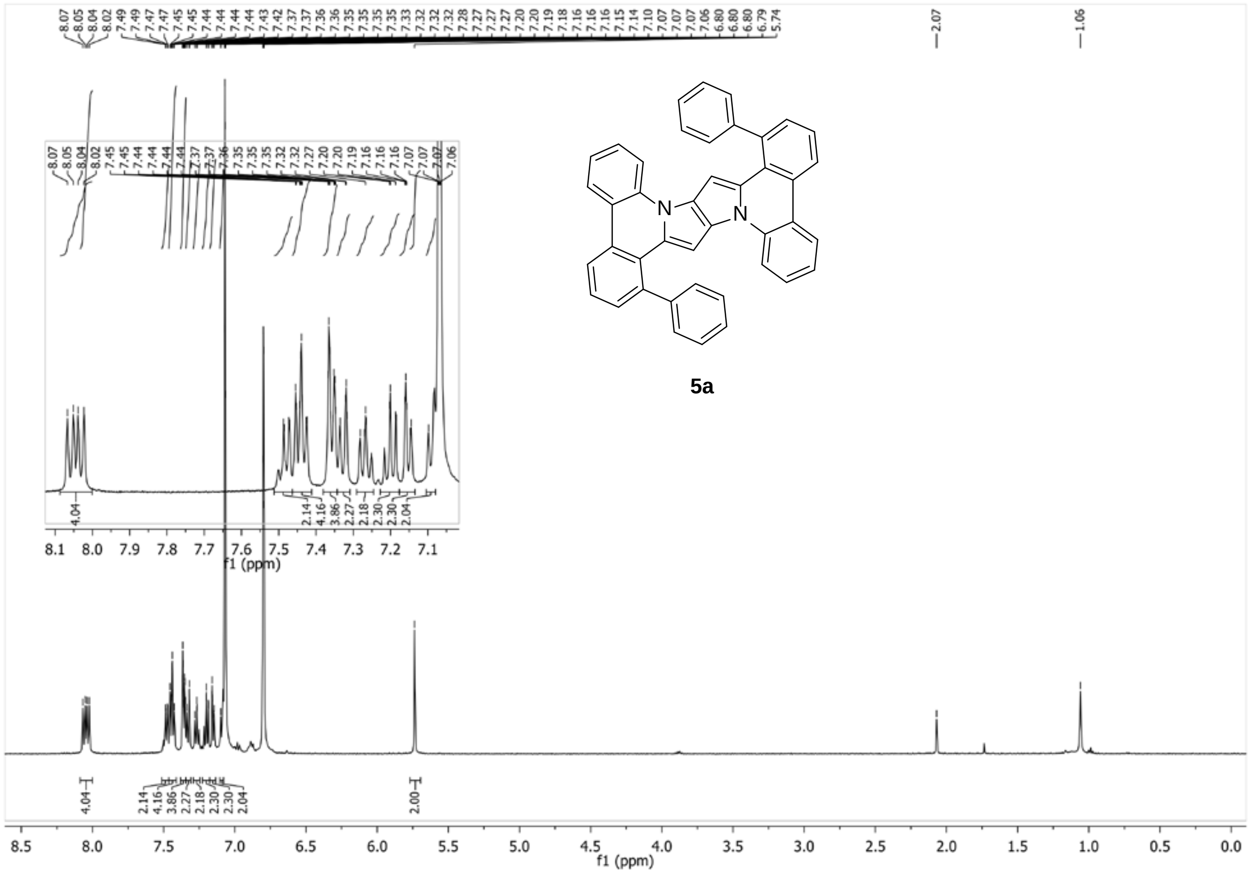

Supplementary Figure 11. ^{1}H NMR spectrum of **5a**.

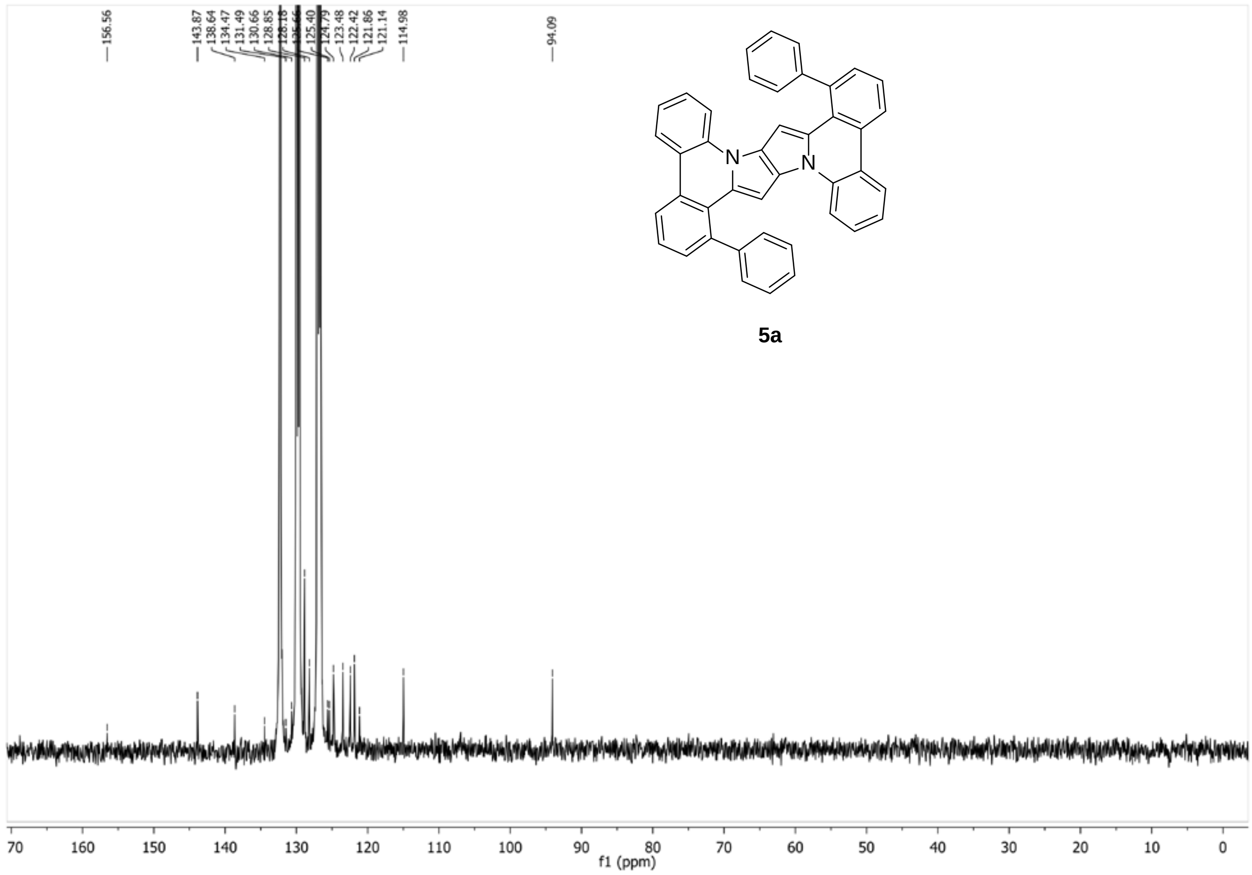

Supplementary Figure 12. ^{13}C NMR spectrum of **5a**.

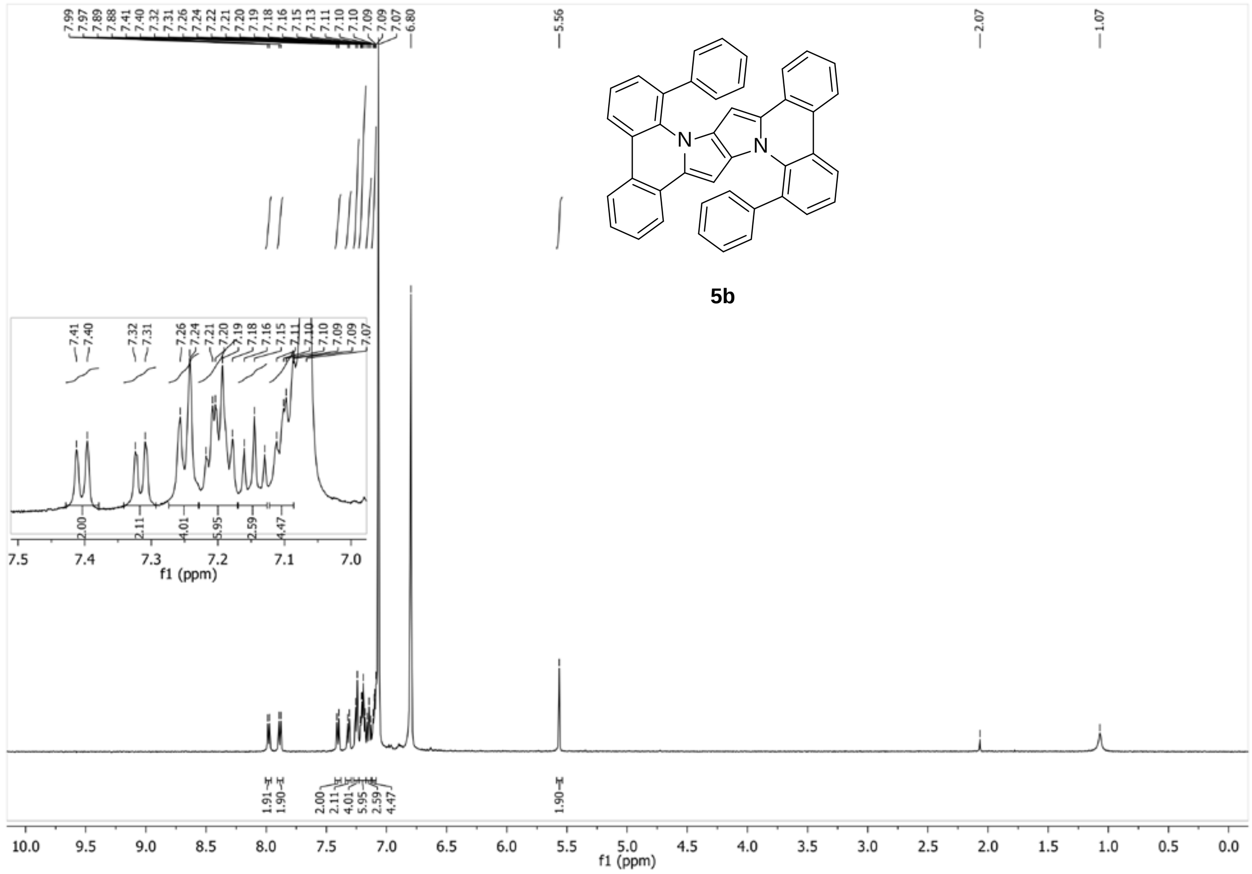

Supplementary Figure 13. ^{1}H NMR spectrum of **5b**.

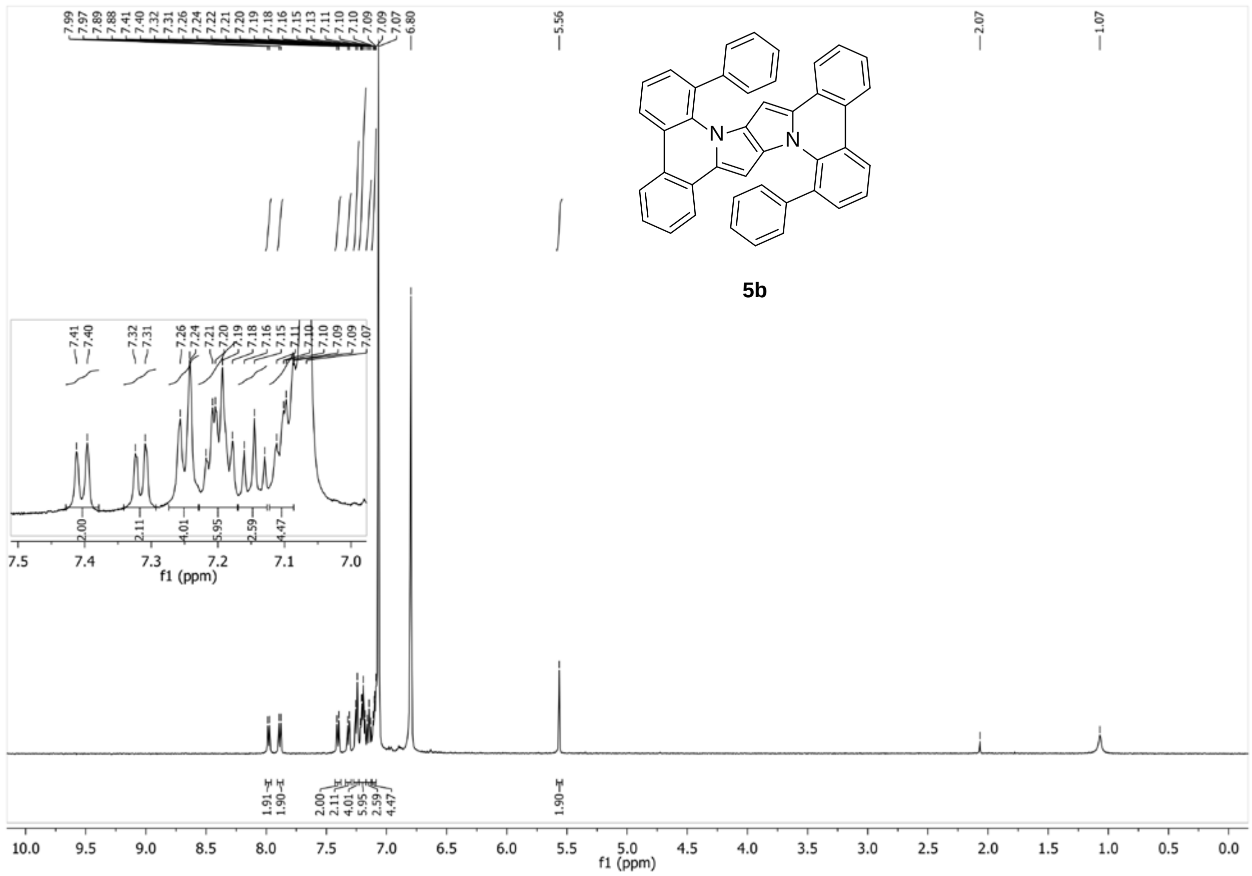

Supplementary Figure 14. ^{13}C NMR spectrum of **5b**.

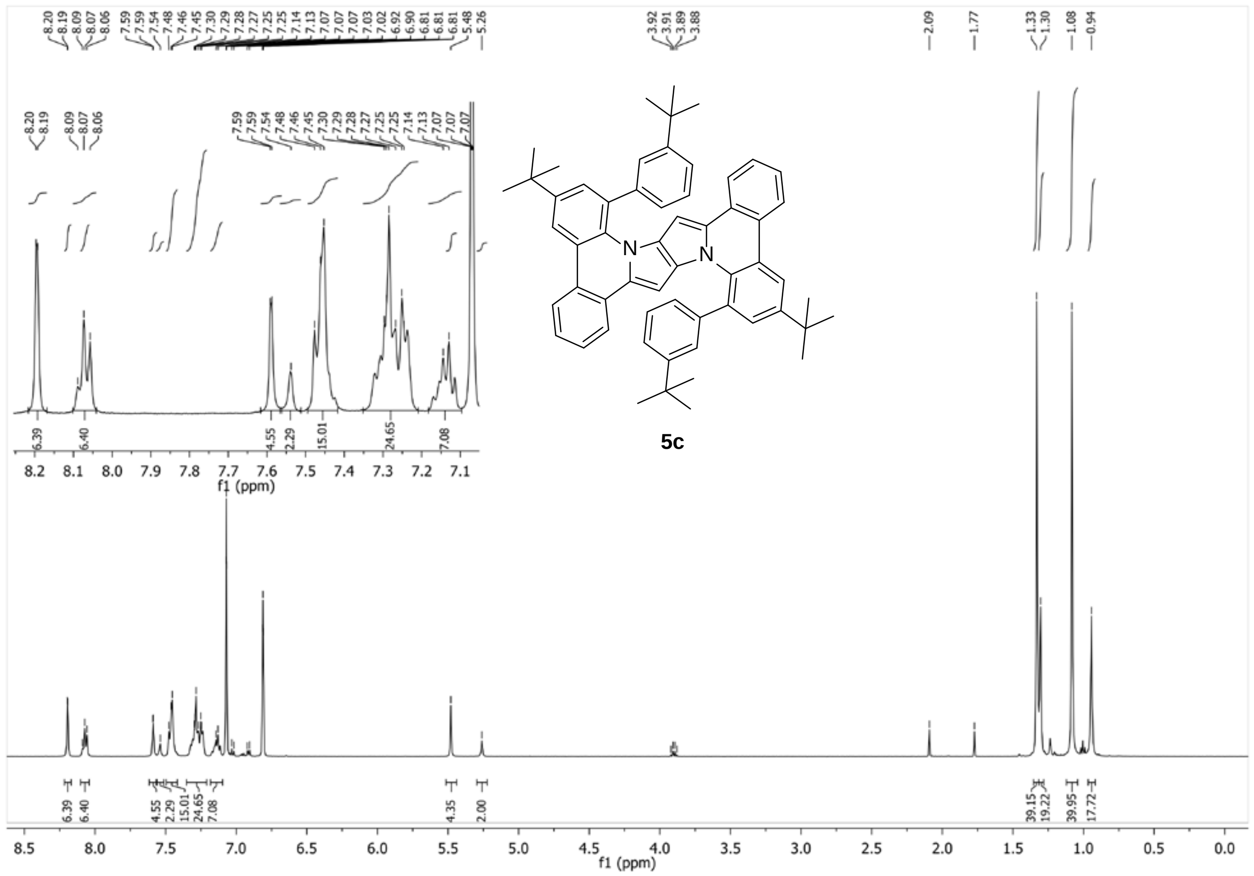

Supplementary Figure 15. ^{1}H NMR spectrum of **5c**.

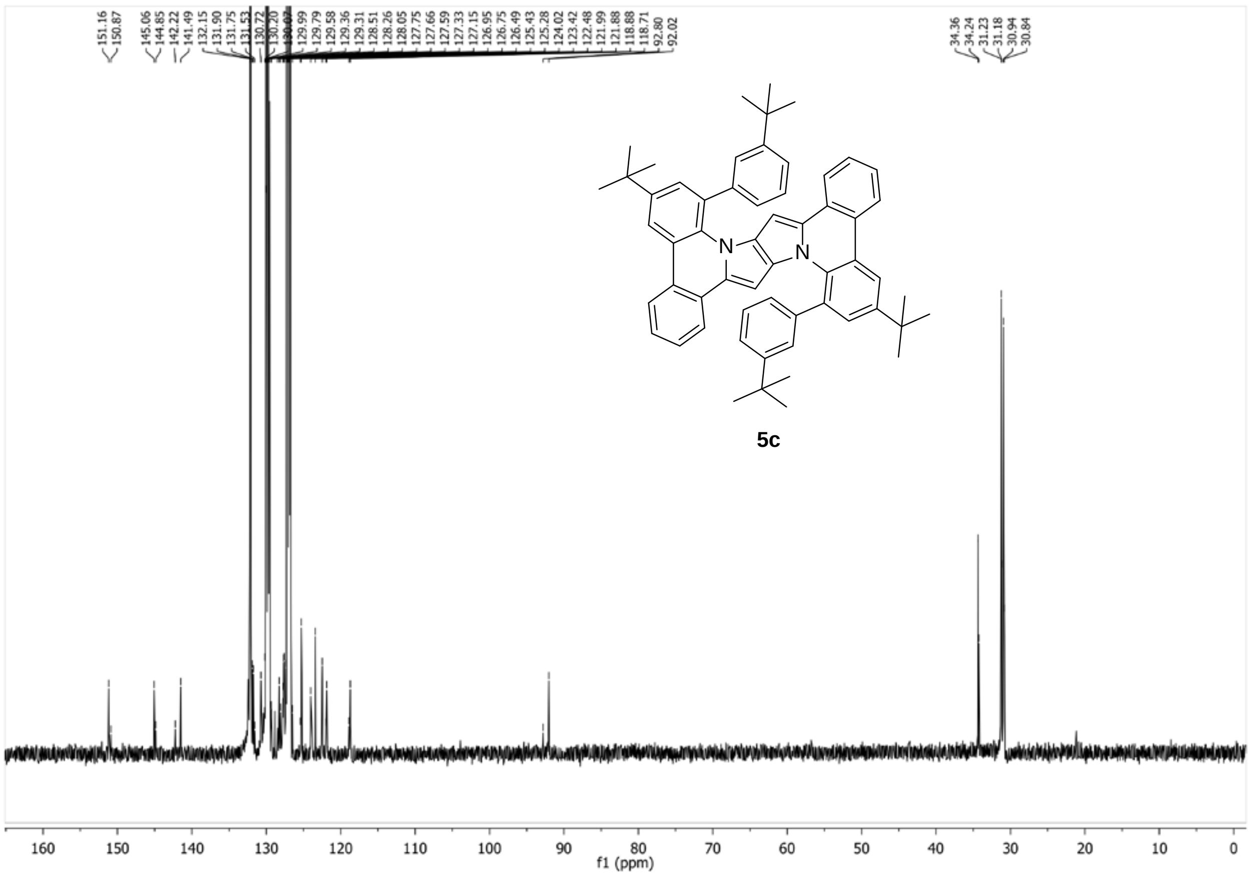

Supplementary Figure 16. ^{13}C NMR spectrum of **5c**.

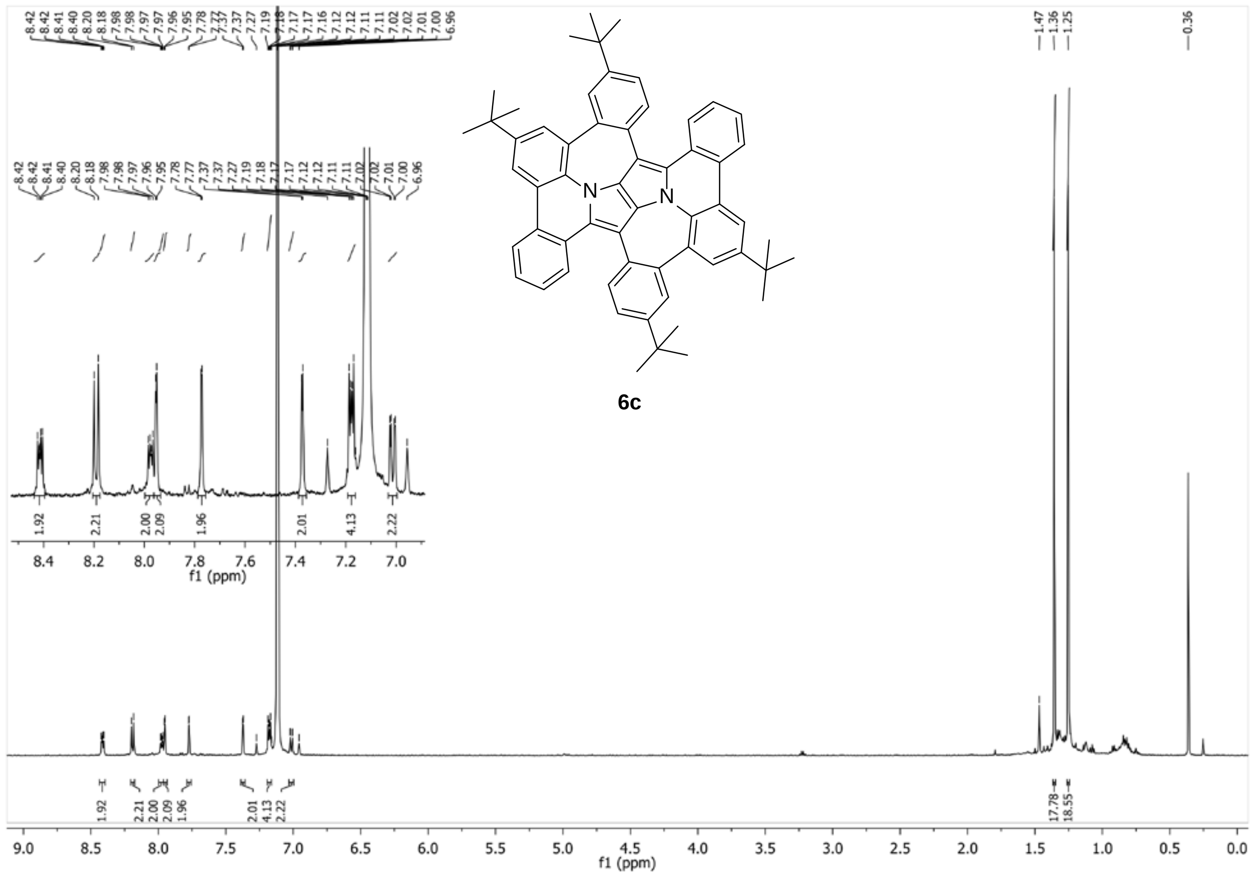

Supplementary Figure 17. ^{1}H NMR spectrum of **6c**.

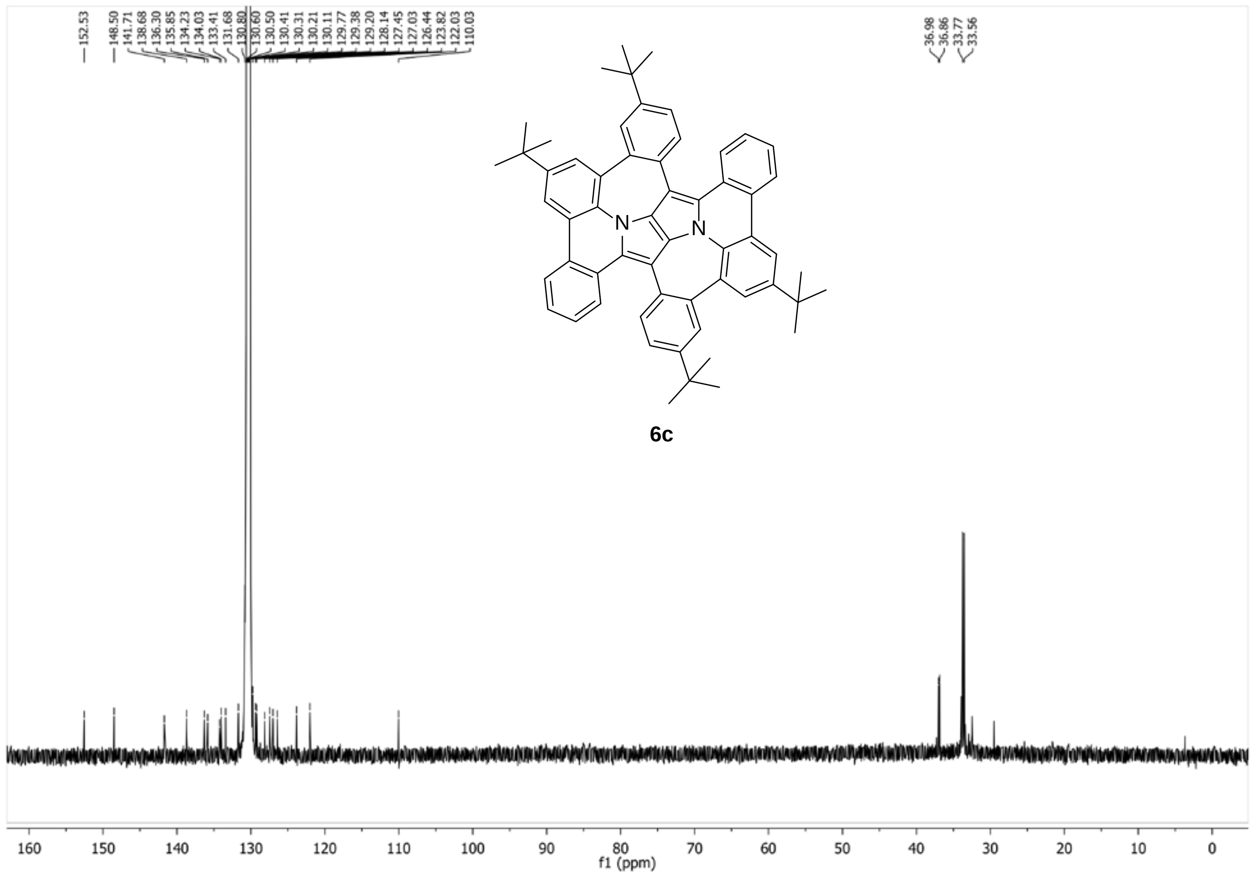

Supplementary Figure 18. ^{13}C NMR spectrum of **6c**.

Supplementary Discussion

1. On-surface reaction of 6a on Ag(111)

Since complete ring closure of **6a** to form **8** is not observed on the Au(111) surface, we tried to induce surface-assisted ring closure on Ag(111), which is a more catalytically active substrate. After sublimation of precursor **6a** under ultra-high vacuum (UHV) conditions onto an atomically clean Ag(111) surface held at room temperature, large-scale STM images (Supplementary Fig. 19a) show the presence of long self-assembled chains and honeycomb islands of **6a**. Supplementary Figures 19b and 19c show high-resolution STM images of the honeycomb islands and chains, respectively. After annealing to 300°C, large-scale STM images (Supplementary Fig. 19d) reveal the presence of mostly chains of **6a** composed of only a few units, few half-closed species (**7a**) and some unknown species. Supplementary Figure 19e shows these structures in detail. Few units corresponding to **7a** are highlighted with red circles. In addition, the unknown species (highlighted with a white circle) presents a much lower apparent height compared to **6a**/**7a**. The identity of these species is not clear, and we propose that they represent decomposed molecules.

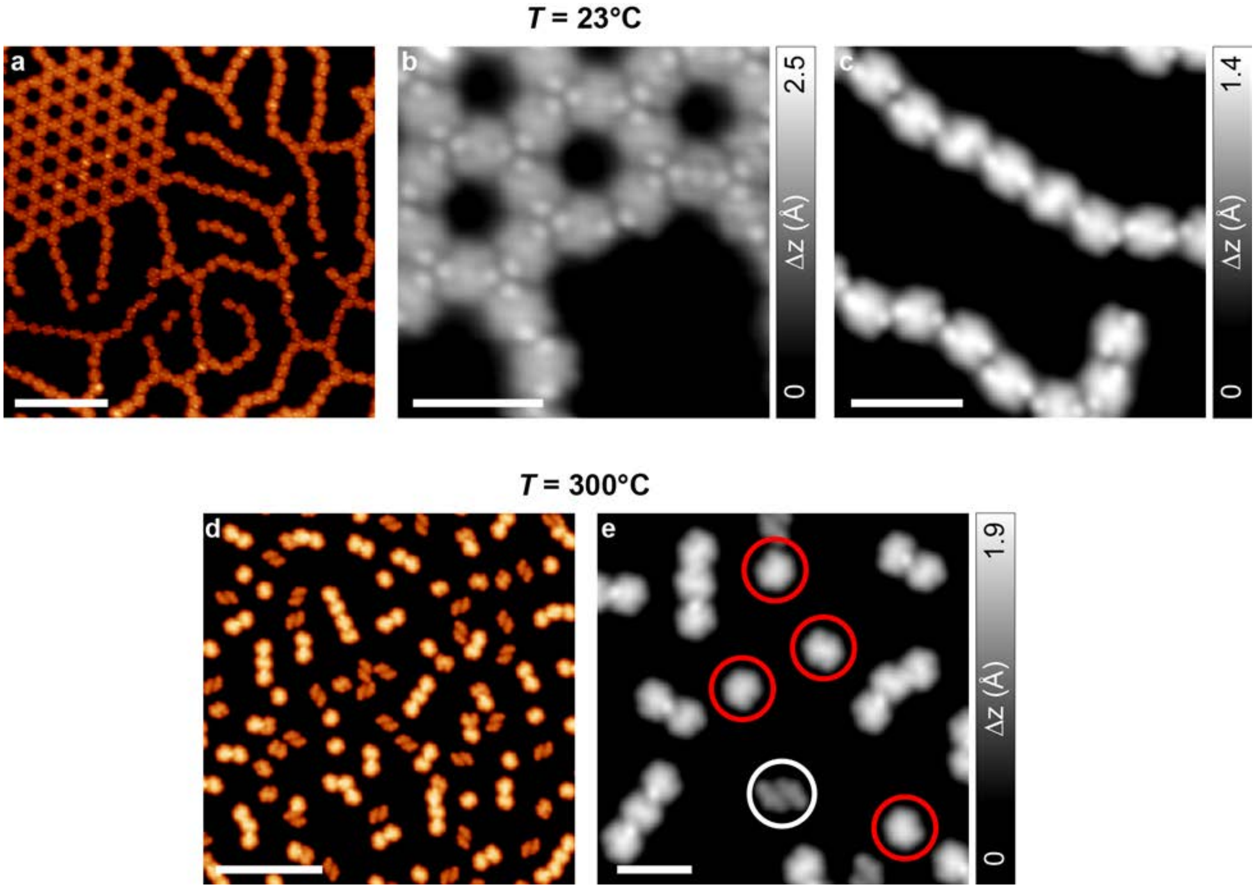

Supplementary Figure 19. On-surface reaction of 6a on Ag(111). (a) Overview STM topography image of the surface after room temperature deposition of **6a**, showing the coexistence of self-assembled chains and honeycomb islands (V = -1 V, I = 40 pA, scale bar: 10 nm). (b) High-resolution STM image of a honeycomb island (V = -1.5 V, I = 50 pA, scale bar: 3 nm). (c) High-resolution STM image of chains (V = -0.1 V, I = 70 pA, scale bar: 3 nm). (d) Overview STM topography image of the surface after annealing to 300°C (V = -1 V, I = 100 pA, scale bar: 10 nm). (e) High-resolution STM image showing coexistence of **6a**, **7a** (red circles) and possibly decomposed molecules (white circle) (V = -1 V, I = 70 pA, scale bar: 3 nm).

2. On-surface reaction of 6a on Cu(111)

After sublimation of precursor **6a** under UHV conditions onto an atomically clean Cu(111) surface held at room temperature, large-scale STM images (Supplementary Fig. 20a) show the presence of self-assembled linear chains presenting a rod-like appearance, as well as individual molecules. Supplementary Figure 20b shows a high-resolution STM image of the self-assembled chains. After annealing to 260°C, intermolecular covalent coupling and decomposition dominate, and ill-defined clusters prevail on the surface. We did not find either **7a** or **8** on the surface (Supplementary Fig. 20c). We conclude that the Cu(111) surface is too active, and promotes decomposition of the molecules.

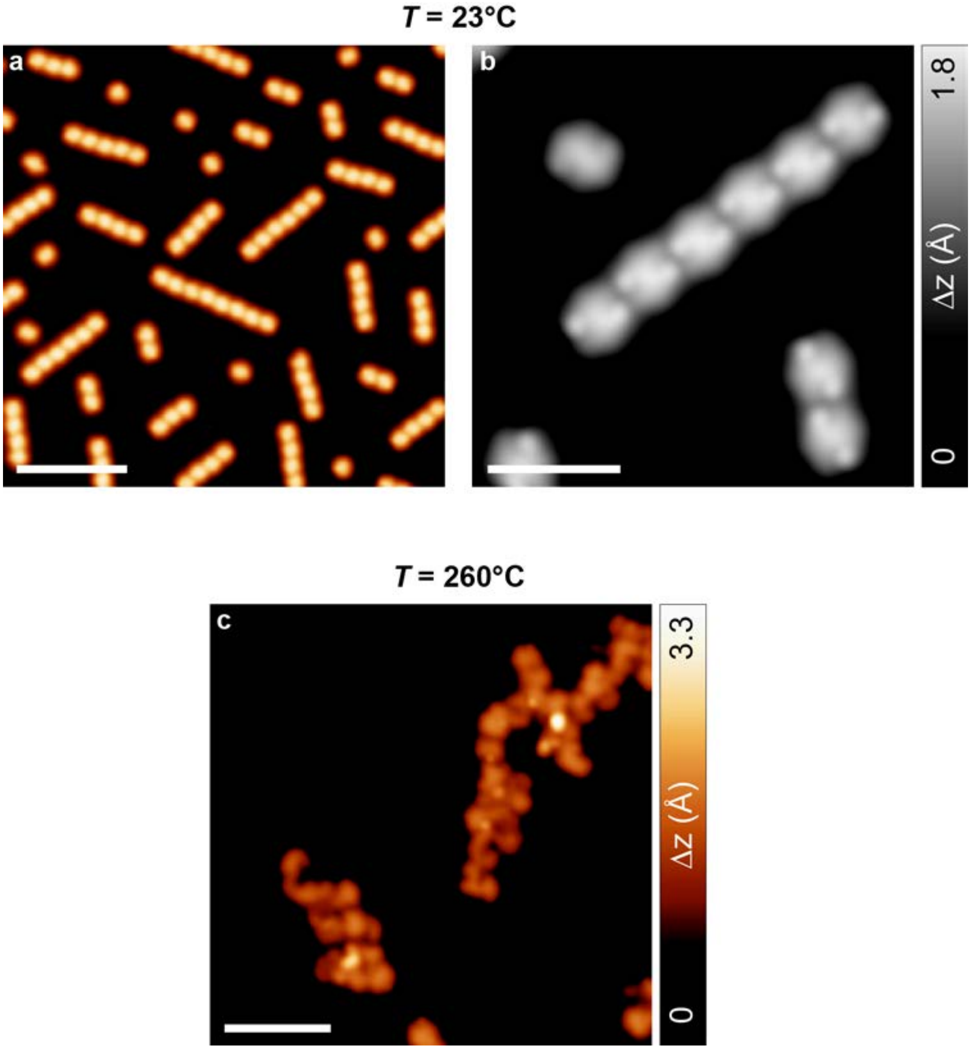

Supplementary Figure 20. On-surface reaction of 6a on Cu(111). (a) Overview STM topography image of the surface after room temperature deposition of **6a**, showing the coexistence of self-assembled linear chains and individual molecules (V = -1 V, I = 40 pA, scale bar: 10 nm). (b) High-resolution STM image of a linear chain of molecules (V = -1 V, I = 70 pA, scale bar: 3 nm). (c) Overview STM topography image of the surface after annealing to 260°C (V = -1 V, I = 60 pA, scale bar: 6 nm), showing mostly decomposed molecules.

3. Comparative annealing of 6a and 6b to 350°C on Au(111)

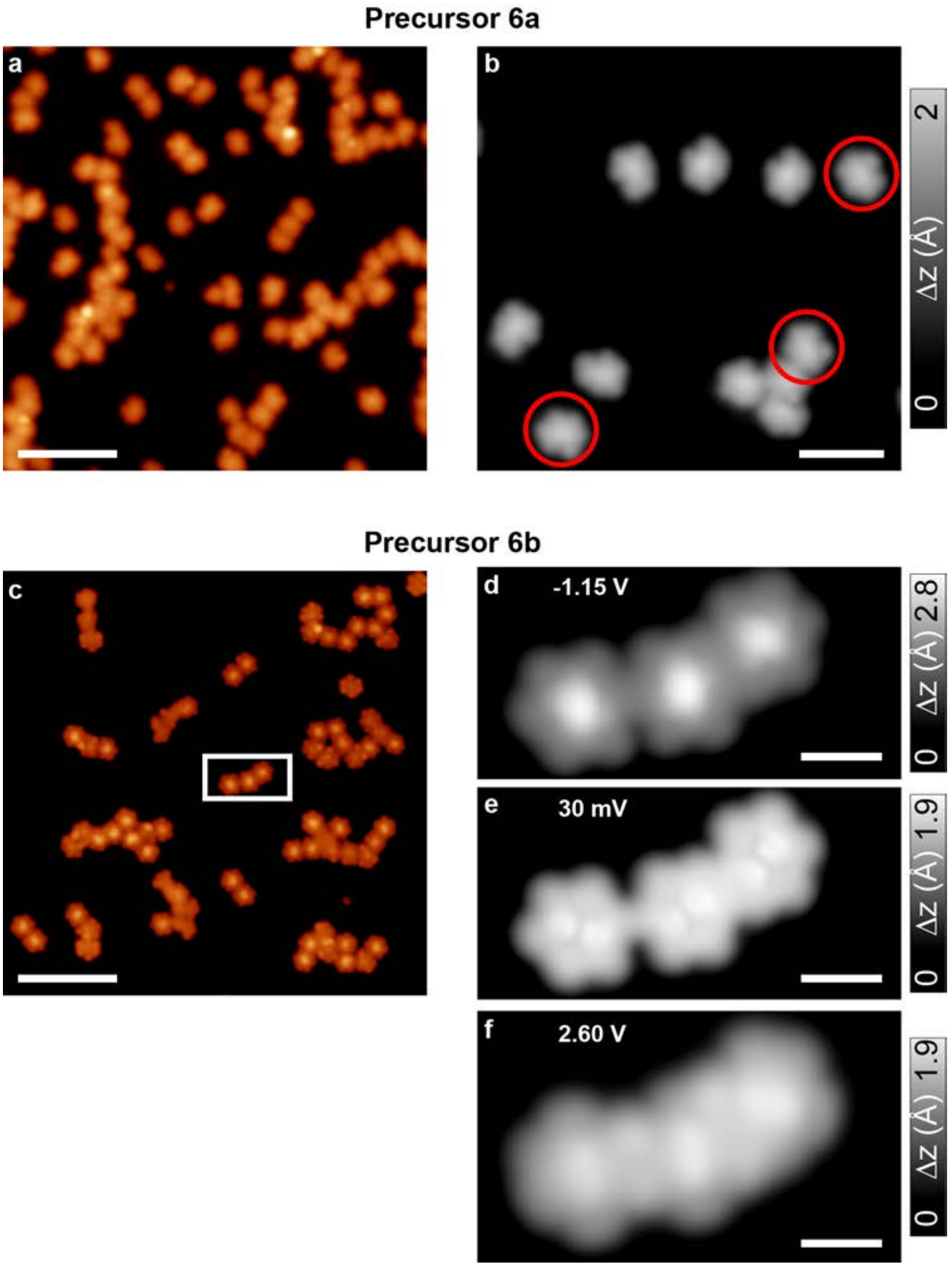

Supplementary Figure 21. Annealing of the precursor molecules to 350°C. (a) Overview STM topography image of **6a/Au(111)** after annealing to 350°C (V = -1 V, I = 40 pA, scale bar: 7 nm). (b) Representative high-resolution STM image of the surface (V = -1 V, I = 70 pA, scale bar: 3 nm). Some units corresponding to **7a** are highlighted with the red circles. However, we did not find **8** on the surface. (c) Overview STM topography image of **6b/Au(111)** after annealing to 350°C (V = -1.15 V, I = 25 pA, scale bar: 7 nm). About 60% of the molecules on the surface correspond to **8**, and are distinguished by appearance of a bright feature at the center of the molecules upon scanning at tunneling bias at or close to -1.15 V (i.e. energetic position of the HOMO). Also, most of the molecules underwent covalent coupling through intermolecular cross-dehydrogenation at 350°C. (d), (e), (f) High-resolution STM images of a covalently connected trimer of **8** highlighted in (c) at indicated tunneling bias voltages at or near to (d) HOMO, (e) gap and (f) LUMO of **8** ((d), (e) I = 50 pA, (f) I = 15 pA; all scale bars: 1 nm).

4. Bowl-opening-up species – 8’

Interestingly, we find a minor amount of a molecular species which first appears after the annealing step to 270°C. Referred to as **8’**, its appearance changes with the tunneling bias voltage. Notably, at positive bias values, the molecule presents a characteristic appearance wherein the periphery of the molecule shows a uniform contrast with the center appearing noticeably dark (Supplementary Fig. 22b and 22c). Similar characteristic feature in STM images of surface-adsorbed buckybowls was reported which corresponded to a bowl-opening-up configuration of the respective buckybowls[1,2]. Furthermore, through UHR-STM images of **8’** (Supplementary Fig. 22d and 22e), we clearly resolve the peripheral hexagonal rings, with considerable loss of contrast on the inner hexagonal and heptagonal rings, and no contrast on the pentagonal rings. These observations strongly hint towards **8’** being the bowl-opening-up variant of **8**. To aid our understanding of the identity of **8’**, we constructed the structure of the bowl-opening-up variant of **8** on Au (111) (shown in Supplementary Fig. 22f and 22g). The corresponding DFT simulated STM images for this conformation (Supplementary Fig. 22h and 22i) show striking agreement with the experimental STM images at both polarities of the tunneling bias voltage, thus verifying our speculation that **8’** is indeed the bowl-opening-up variant of **8.** The factors stabilizing this conformation on the surface are presently not known.

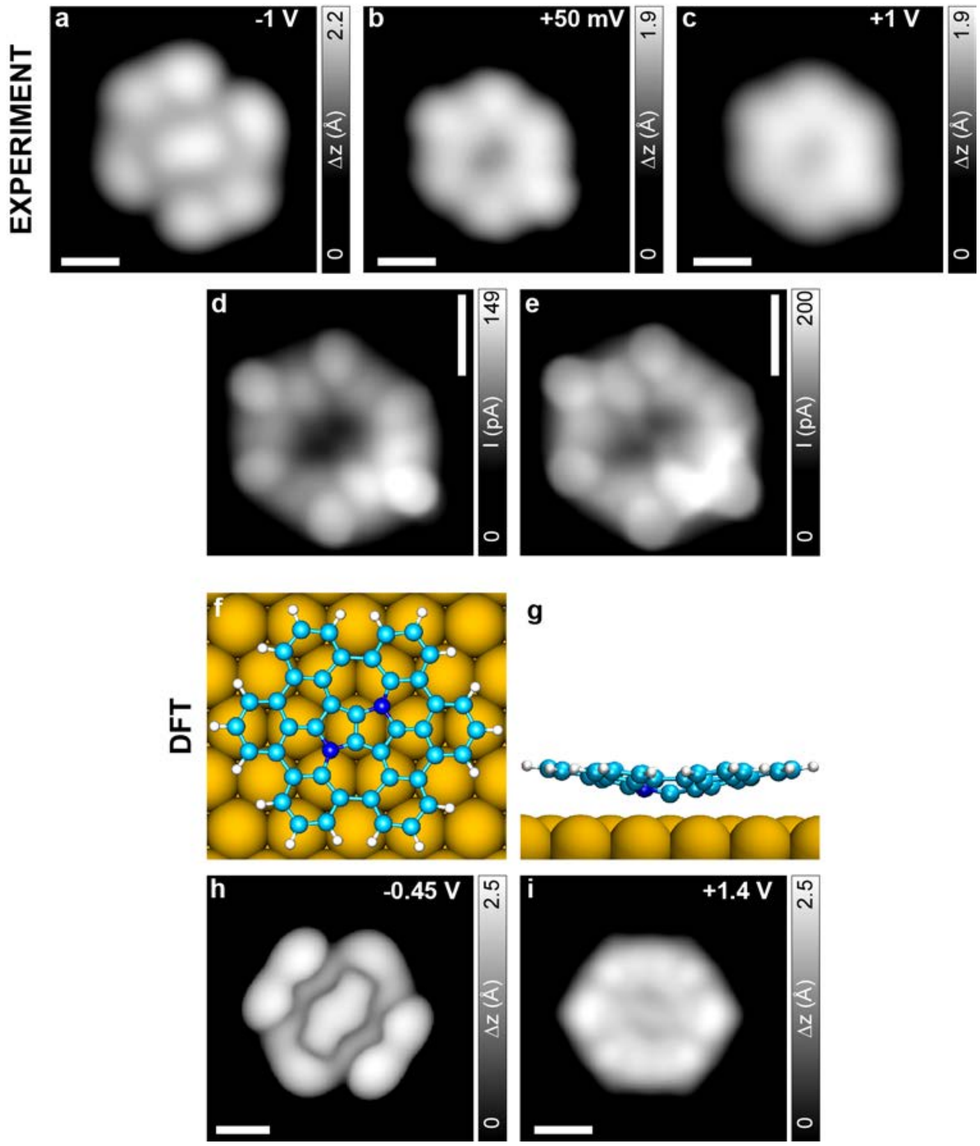

Supplementary Figure 22. Identification of 8'. (a), (b), (c) High-resolution STM images of **8'** acquired with a CO tip at different tunneling bias voltages as indicated on the respective figures (I = 50 pA). (d), (e) Corresponding UHR-STM images at varying tip-sample distances (Open feedback parameters: V = 5 mV, I = 50 pA; (d) Δz = -35 pm, (e) Δz = -50 pm). (f), (g) Top and side views of the geometry used for simulating the STM images. (h), (i) Corresponding DFT simulated STM images at indicated bias values. All scale bars: 0.5 nm.

5. DFT-calculated molecular orbitals of 8

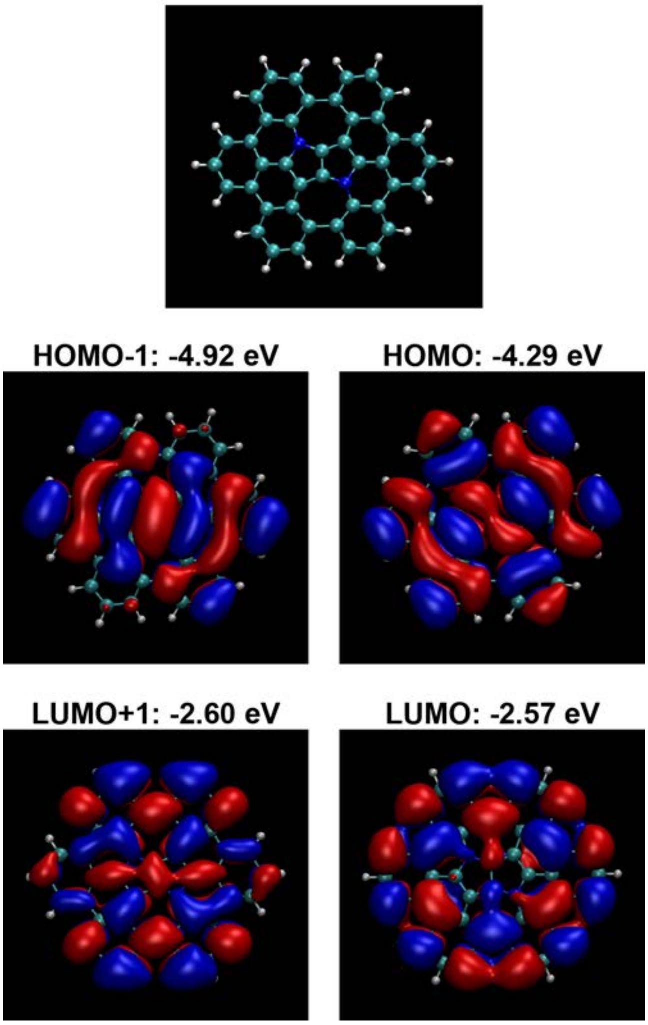

Supplementary Figure 23. Calculated gas-phase frontier molecular orbitals of 8. DFT-calculated chemical structure model and gas-phase frontier molecular orbitals of **8**. Energetic position of the orbitals are stated with respect to the vacuum level (E = 0 eV). For each figure, the view corresponds to the convex (i.e. 'outer') face of the molecule.

6. Additional dI/dV spectra of 8

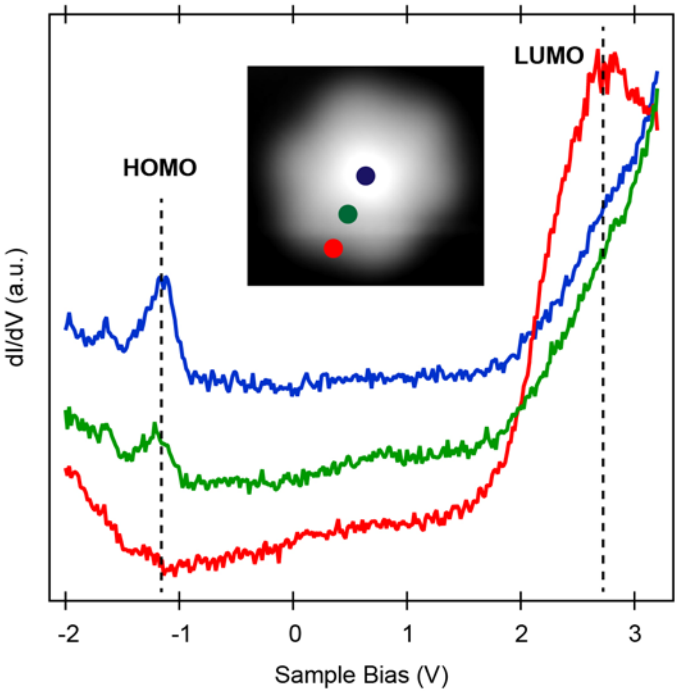

Supplementary Figure 24. dI/dV spectra of 8. Set of dI/dV spectra showing the evolution of frontier orbital peaks from the center towards the periphery of **8**. The spectra are vertically shifted for clarity. Inset: STM topography image of **8** (V = -2 V, I = 40 pA). The colored circles mark the positions at which the corresponding STS spectra were acquired. Open feedback parameters for the STS spectra: V = -2 V, I = 40 pA, V_{rms} = 20 mV, f = 860 Hz.

7. Gas-phase bowl-inversion energetics of 8

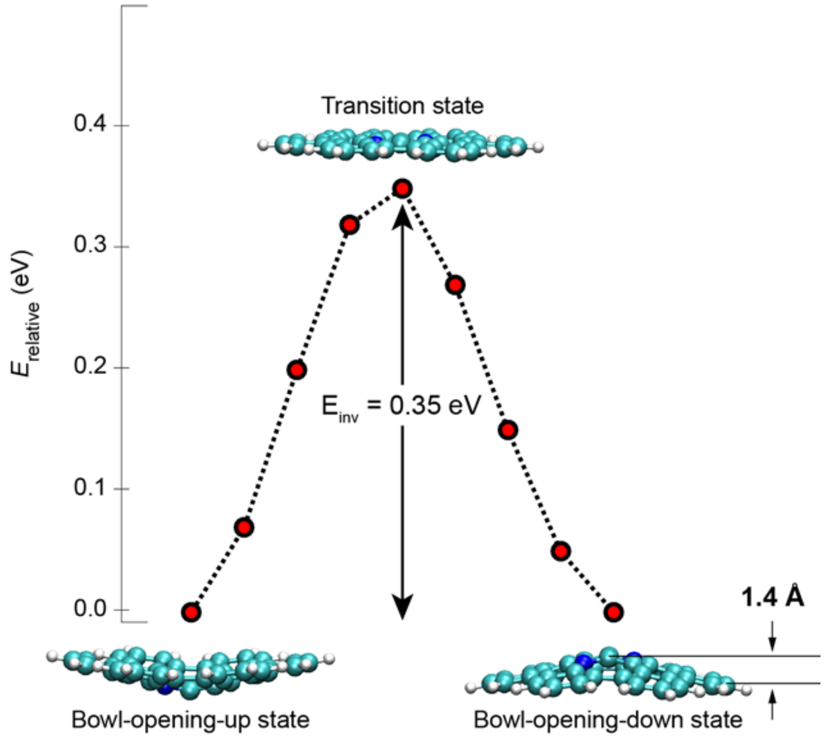

Supplementary Figure 25. The figure displays the DFT nudged elastic band (DFT-NEB) calculated relative energetics of bowl-inversion of **8** in the gas phase as the system proceeds from an initial bowl-opening-up state to the final bowl-opening-down state *via* a planar transition state. The calculated inversion energy barrier (E_{inv}), defined as the difference in energies between the ground states (bowl-opening-up/bowl-opening-down) and the highest-energy transition state (planar state), amounts to 0.35 eV - comparable to the calculated gas-phase inversion energy barrier of corannulene (0.50 eV)[3] and lower than that of sumanene (1.00 eV)[4]. The calculated bowl-depth of **8** in gas-phase amounts to 1.40 Å, as compared to 1.20 Å on Au(111).

8. Conformational stability of 8 on Au(111)

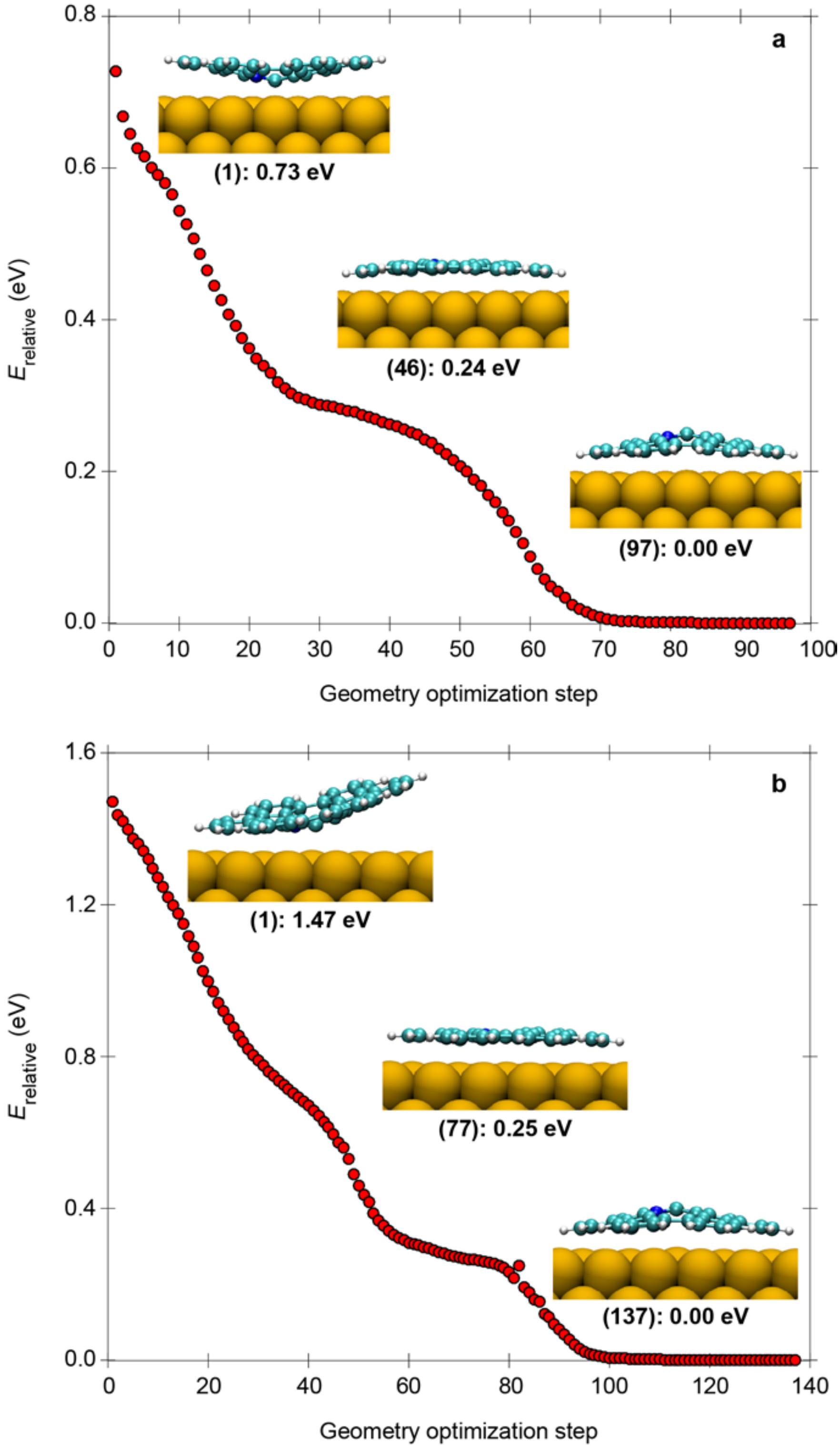

Supplementary Figure 26. Relative stability of on-surface conformations of 8 on Au(111) during two geometry optimization procedures. Plot depicting the DFT-based energies ($E_{relative}$) in eV of various conformations of **8** on Au(111) relative to the bowl-opening-down conformation during two geometry optimization procedures. Insets in each plot show three different conformations with their optimization step numbers (in parentheses) and corresponding relative energies. **(a)** (1): initial bowl-opening-up conformation, (46): intermediate approximately-planar conformation, and (97): final converged bowl-opening-down conformation; **(b)** (1): initial *tilted*

bowl-opening-up conformation, (77): intermediate approximately-planar conformation, and (137): final converged bowl-opening-down conformation. Within our geometry optimization procedures, employing the popular scheme proposed by Grimme *et al.* to account for van der Waals interaction[5], we find that the most stable conformation of **8** on Au(111) is the bowl-opening-down – a theoretical result that is corroborated in our experiments. The observation of a minor amount of bowl-opening-up species in the experiments (Supplementary Fig. 22) suggests that the empirical van der Waals parameter employed in our study is stronger than the optimum, but nevertheless provides an overall faithful and correct trend. Two different starting geometries (i.e. bowl-opening-up and *tilted* bowl-opening-up) were chosen for the geometry optimization procedures in order to make sure that the choice of initial geometry does not critically influence the final optimized result.

Note that while the bowl-inversion process in the gas-phase has a finite energy barrier of 0.35 eV (Supplementary Fig. 25), geometry optimization procedures on Au(111) resulting in a final bowl-opening-down geometry from initial bowl-opening-up geometries show no energy barriers. This highlights the crucial role of molecule-substrate interaction in determining the conformation of **8** on the surface.

9. Adsorption energetics of 8 on Au(111)

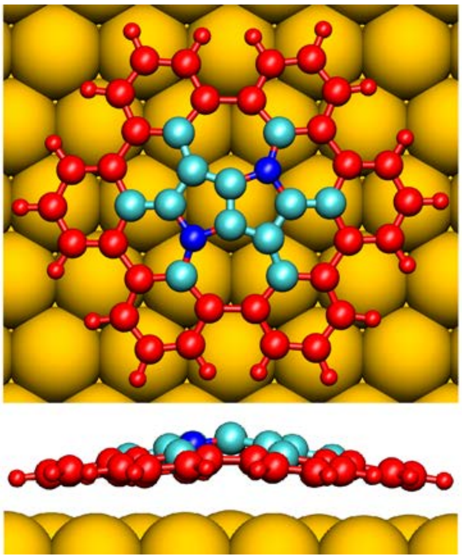

E_{ads}	-4.10 eV
$E_{vdW, total}$	-4.62 eV
$E_{vdW, rim}$	-3.81 eV
$E_{vdW, center}$	-1.08 eV

Supplementary Figure 27. (left panel) Top and side views of the DFT-optimized structure of **8** on Au(111). The colors highlight the rim carbon and hydrogen atoms (red) and the central carbon and nitrogen atoms (cyan and blue, respectively). (right panel) Table displaying the DFT-calculated values of total adosorption energy of **8** on Au(111) (**E_{ads}**), contribution of pure van der Waals attraction between **8** and Au(111) to E_{ads} (**$E_{vdW, total}$**), contribution of the rim atoms of **8** to $E_{vdW, total}$ (**$E_{vdW, rim}$**), and contribution of the central atoms of **8** to $E_{vdW, total}$ (**$E_{vdW, center}$**).

Supplmentary Figure26, which shows a barrierless evolution of bowl-opening-up conformations to bowl-opening-down conformation, highlights that the molecule-substrate interaction is crucial in **8** adopting the bowl-opening-down conformation. To shed light on the possible origin of the unique bowl-opening-down conformation of **8** on Au(111), we investigate the DFT-calculated adsorption energetics of **8** on Au(111). We start with calculating the total adsorption energy of **8** on Au(111), amounting to E_{ads} = -4.10 eV (Supplementary Fig. 27, right panel) obtained after subtracting from the total energy of the equilibrium adsorption geometry, the energy of the equilibrium geometry of **8** in the gas-phase and the energy of the equilibrium geometry of the clean Au slab. The contribution of pure van der Waals (vdW) attraction between **8** and Au(111) ($E_{vdW, total}$) to E_{ads} amounts to -4.62 eV. The 0.52 eV lower value of $E_{vdW, total}$ compared to E_{ads} stems from the contribution of Pauli repulsion to the E_{ads} term. Further on, we estimate the vdW energy contribution of the rim atoms (colored red in Supplementary Fig. 27, left panel), $E_{vdW, rim}$, and the central atoms (colored in cyan and blue in Supplementary Fig. 27, left panel), $E_{vdW, center}$, of **8** to $E_{vdW, total}$. Our analysis leads to $E_{vdW, rim}$ = -3.81 eV and $E_{vdW, center}$ = -1.08 eV. There is a slight discrepancy between the vdW energy terms as $E_{vdW, total}$ = -4.62 eV does not equal $E_{vdW, rim}$ + $E_{vdW, center}$ = -4.89 eV, which arises because in calculating the energetic contributions of parts of **8** to the total vdW energy, i.e. $E_{vdW, rim}$ ($E_{vdW, center}$), the absence of the corresponding central (rim) atoms leads to renormalization of the coordination dependent vdW parameters in the Grimme model[5] for the rim and central atoms. However, since the magnitude of the discrepancy term (0.28 eV) is small compared to $E_{vdW, rim}$ and $E_{vdW, center}$; our analysis still allows us to make important inferences, at least on a qualitative level, which is explained in the next paragraph.

From the respective values of $E_{vdW, rim}$ and $E_{vdW, center}$, it is seen that the rim atoms contribute a much higher fraction to the total vdW energy than the central atoms. In a bowl-opening-up conformation, where the rim atoms would be farther from the surface compared to the bowl-opening-down conformation, the resulting loss of vdW energy may not be compensated by the gain in vdW energy due to the increased proximity of the central atoms to the surface, which may explain the preference

of **8** to adopt a bowl-opening-down conformation on the surface. We note that since the bowl-opening-up conformation is not a stable conformation on the surface (Supplementary Fig. 26), it is not possible to directly compare the relative change in vdW energy between the two conformations on the surface. Our qualitative argument serves to illustrate that the driving force for **8** to adopt a bowl-opening-down conformation is the maximization of vdW interaction between **8** and the underlying Au(111) substrate.

Supplementary References

1. Parschau, M. *et al.* Buckybowls on Metal Surfaces: Symmetry Mismatch and Enantiomorphism of Corannulene on Cu(110). *Angew. Chem. Int. Ed.* **46,** 8258–8261 (2007).
2. Stöckl, Q. S., Hsieh, Y.-C., Mairena, A., Wu, Y.-T. & Ernst, K.-H. Aggregation of C70-Fragment Buckybowls on Surfaces: π–H and π–π Bonding in Bowl Up-Side-Down Ensembles. *J. Am. Chem. Soc.* **138,** 6111–6114 (2016).
3. Seiders, T. J., Baldridge, K. K., Grube, G. H. & Siegel, J. S. Structure/Energy Correlation of Bowl Depth and Inversion Barrier in Corannulene Derivatives: Combined Experimental and Quantum Mechanical Analysis. *J. Am. Chem. Soc.* **123,** 517–525 (2001).
4. Jaafar, R. *et al.* Bowl Inversion of Surface-Adsorbed Sumanene. *J. Am. Chem. Soc.* **136,** 13666–13671 (2014).
5. Grimme, S., Antony, J., Ehrlich, S. & Krieg, H. A consistent and accurate ab initio parametrization of density functional dispersion correction (DFT-D) for the 94 elements H-Pu. *J. Chem. Phys.* **132,** 154104 (2010).
