# Supplementary material for: On-surface synthesis of a nitrogen-embedded buckybowl with inverse Stone–Thrower–Wales topology
Source: Nat Commun. 2018 Apr 30;9:1714. doi: 10.1038/s41467-018-04144-5 (PMC5928119; doi:10.1038/s41467-018-04144-5)
Supplement: Supplementary file 2 — Descriptions of Additional Supplementary Files [file 41467_2018_4144_MOESM2_ESM.pdf]

Descriptions of Additional Supplementary Files

File Name: Supplementary Dataset 1
Description: checkCIF/PLATON report for compound 4b

File Name: Supplementary Dataset 2
Description: CIF file for compound 4b

File Name: Supplementary Dataset 3
Description: checkCIF/PLATON report for compound 6c

File Name: Supplementary Dataset 4
Description: CIF file for compound 6c
