# Supplementary material for: On-surface synthesis of a nitrogen-embedded buckybowl with inverse Stone–Thrower–Wales topology
Source: Nat Commun. 2018 Apr 30;9:1714. doi: 10.1038/s41467-018-04144-5 (PMC5928119; doi:10.1038/s41467-018-04144-5)

# checkCIF/PLATON report

Structure factors have been supplied for datablock(s) MK300_A_0m

THIS REPORT IS FOR GUIDANCE ONLY. IF USED AS PART OF A REVIEW PROCEDURE FOR PUBLICATION, IT SHOULD NOT REPLACE THE EXPERTISE OF AN EXPERIENCED CRYSTALLOGRAPHIC REFEREE.

No syntax errors found. CIF dictionary Interpreting this report

# Datablock: MK300_A_0m

---

```
Bond precision:  C-C = 0.0092 A          Wavelength=1.54178

Cell:            a=13.3201(6)    b=15.4521(8)       c=16.7606(8)
                 alpha=90        beta=108.181(3)    gamma=90
Temperature:     296 K

                Calculated                Reported
Volume          3277.5(3)                 3277.5(3)
Space group     P 21/c                    P 21/c
Hall group      -P 2ybc                   -P 2ybc
Moiety formula  C42 H28 Br2 N2            C42 H28 Br2 N2
Sum formula     C42 H28 Br2 N2            C42 H28 Br2 N2
Mr              720.46                    720.48
Dx,g cm-3       1.460                     1.460
Z               4                         4
Mu (mm-1)       3.386                     3.386
F000            1456.0                    1456.0
F000'           1453.69
h,k,lmax        16,18,20                  16,18,20
Nref            6032                      6005
Tmin,Tmax       0.452,0.708               0.322,0.724
Tmin'           0.201

Correction method= # Reported T Limits: Tmin=0.322 Tmax=0.724
AbsCorr = NUMERICAL

Data completeness= 0.996          Theta(max)= 68.428

R(reflections)= 0.0670( 4041)     wR2(reflections)= 0.2012( 6005)

S = 1.048                  Npar= 424
```

---

The following ALERTS were generated. Each ALERT has the format
**test-name_ALERT_alert-type_alert-level**.
Click on the hyperlinks for more details of the test.

## Alert level B

```
PLAT230_ALERT_2_B Hirshfeld Test Diff for    C13    --  C18     ..       16.4 s.u.
```

## Alert level C

```
RINTA01_ALERT_3_C  The value of Rint is greater than 0.12
            Rint given   0.127
PLAT230_ALERT_2_C Hirshfeld Test Diff for    Br2    --  C14     ..        5.7 s.u.
PLAT230_ALERT_2_C Hirshfeld Test Diff for    C17    --  C18     ..        7.0 s.u.
PLAT341_ALERT_3_C Low Bond Precision on  C-C Bonds ...............    0.00924 Ang.
PLAT369_ALERT_2_C Long    C(sp2)-C(sp2) Bond  C13    -   C18    ..       1.53 Ang.
PLAT911_ALERT_3_C Missing # FCF Refl Between THmin & STh/L=  0.600         10 Report
PLAT978_ALERT_2_C Number C-C Bonds with Positive Residual Density.          0 Info
```

## Alert level G

```
PLAT020_ALERT_3_G The value of Rint is greater than 0.12 .........      0.127 Report
PLAT083_ALERT_2_G SHELXL Second Parameter in WGHT  Unusually Large       6.15 Why ?
PLAT164_ALERT_4_G Nr. of Refined C-H H-Atoms in Heavy-Atom Struct.          2 Note
PLAT335_ALERT_2_G Check Large C6 Ring    C-C Range C13    -C18           0.18 Ang.
PLAT432_ALERT_2_G Short Inter X...Y Contact  Br2    ..  C40     ..       3.31 Ang.
PLAT432_ALERT_2_G Short Inter X...Y Contact  Br2    ..  C41     ..       3.35 Ang.
PLAT912_ALERT_4_G Missing # of FCF Reflections Above STh/L=  0.600         18 Note
```

0 **ALERT level A** = Most likely a serious problem - resolve or explain
1 **ALERT level B** = A potentially serious problem, consider carefully
7 **ALERT level C** = Check. Ensure it is not caused by an omission or oversight
7 **ALERT level G** = General information/check it is not something unexpected

0 ALERT type 1 CIF construction/syntax error, inconsistent or missing data
9 ALERT type 2 Indicator that the structure model may be wrong or deficient
4 ALERT type 3 Indicator that the structure quality may be low
2 ALERT type 4 Improvement, methodology, query or suggestion
0 ALERT type 5 Informative message, check

It is advisable to attempt to resolve as many as possible of the alerts in all categories. Often the minor alerts point to easily fixed oversights, errors and omissions in your CIF or refinement strategy, so attention to these fine details can be worthwhile. In order to resolve some of the more serious problems it may be necessary to carry out additional measurements or structure refinements. However, the purpose of your study may justify the reported deviations and the more serious of these should normally be commented upon in the discussion or experimental section of a paper or in the "special_details" fields of the CIF. checkCIF was carefully designed to identify outliers and unusual parameters, but every test has its limitations and alerts that are not important in a particular case may appear. Conversely, the absence of alerts does not guarantee there are no aspects of the results needing attention. It is up to the individual to critically assess their own results and, if necessary, seek expert advice.

**Publication of your CIF in IUCr journals**

A basic structural check has been run on your CIF. These basic checks will be run on all CIFs submitted for publication in IUCr journals (*Acta Crystallographica*, *Journal of Applied Crystallography*, *Journal of Synchrotron Radiation*); however, if you intend to submit to *Acta Crystallographica Section C* or *E* or *IUCrData*, you should make sure that full publication checks are run on the final version of your CIF prior to submission.

**Publication of your CIF in other journals**

Please refer to the *Notes for Authors* of the relevant journal for any special instructions relating to CIF submission.

---

**PLATON version of 13/08/2017; check.def file version of 27/07/2017**

**Datablock MK300_A_0m** - ellipsoid plot

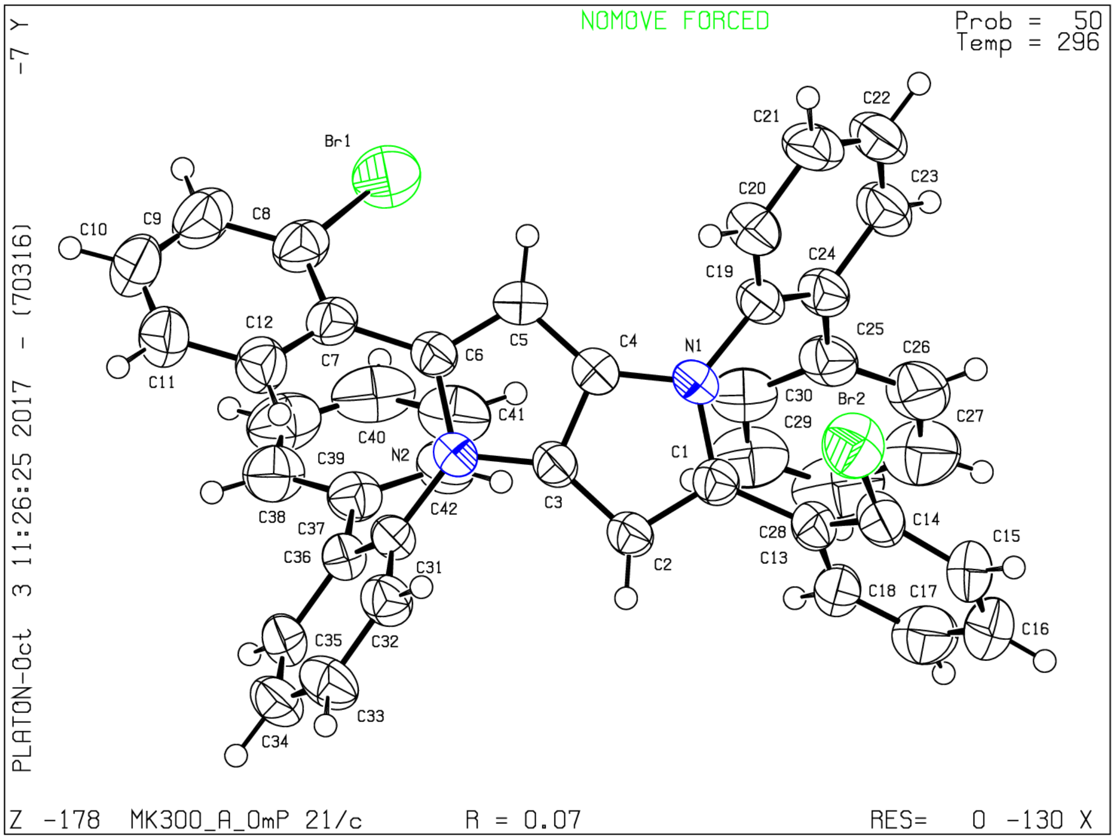

Supplement: Supplementary file 3 — Supplementary Data 1 [file 41467_2018_4144_MOESM3_ESM.pdf]
