# Supplementary material for: On-surface synthesis of a nitrogen-embedded buckybowl with inverse Stone–Thrower–Wales topology
Source: Nat Commun. 2018 Apr 30;9:1714. doi: 10.1038/s41467-018-04144-5 (PMC5928119; doi:10.1038/s41467-018-04144-5)

checkCIF/PLATON report

Structure factors have been supplied for datablock(s) MK460_0m

THIS REPORT IS FOR GUIDANCE ONLY. IF USED AS PART OF A REVIEW PROCEDURE FOR PUBLICATION, IT SHOULD NOT REPLACE THE EXPERTISE OF AN EXPERIENCED CRYSTALLOGRAPHIC REFEREE.

No syntax errors found. CIF dictionary Interpreting this report

Datablock: MK460_0m

```
Bond precision:  C-C = 0.0129 A          Wavelength=1.54178

Cell:           a=13.8828(12)    b=10.8415(9)      c=19.0960(17)
                alpha=90         beta=90.513(5)    gamma=90
Temperature:    296 K

                Calculated                 Reported
Volume          2874.0(4)                  2874.0(4)
Space group     P 21/n                     P 21/n
Hall group      -P 2yn                     -P 2yn
Moiety formula  C58 H54 N2, 2(C6 H14)      C58 H54 N2, 2(C6 H14)
Sum formula     C70 H82 N2                 C70 H82 N2
Mr              951.38                     951.37
Dx,g cm-3       1.099                      1.099
Z               2                          2
Mu (mm-1)       0.466                      0.466
F000            1032.0                     1032.0
F000'           1034.52
h,k,lmax        15,12,21                   12,11,21
Nref            4306                       3163
Tmin,Tmax       0.897,0.930                0.975,0.991
Tmin'           0.824

Correction method= # Reported T Limits: Tmin=0.975 Tmax=0.991
AbsCorr = NUMERICAL

Data completeness= 0.735          Theta(max)= 60.294

R(reflections)= 0.1110( 1649)     wR2(reflections)= 0.3345( 3163)

S = 1.098                  Npar= 336
```

The following ALERTS were generated. Each ALERT has the format
test-name_ALERT_alert-type_alert-level.
Click on the hyperlinks for more details of the test.

Alert level B

```
THETM01_ALERT_3_B  The value of sine(theta_max)/wavelength is less than 0.575
            Calculated sin(theta_max)/wavelength =    0.5634
PLAT031_ALERT_4_B Refined Extinction Parameter Within Range ......      2.000 Sigma
PLAT201_ALERT_2_B Isotropic non-H Atoms in Main Residue(s) .......          4 Report
PLAT340_ALERT_3_B Low Bond Precision on  C-C Bonds ...............    0.01291 Ang.
PLAT911_ALERT_3_B Missing FCF Refl Between Thmin & STh/L=    0.563        886 Report
```

Alert level C

```
REFNR01_ALERT_3_C  Ratio of reflections to parameters is < 10 for a
            centrosymmetric structure
            sine(theta)/lambda              0.5634
            Proportion of unique data used    1.0000
            Ratio reflections to parameters   9.4137
PLAT018_ALERT_1_C _diffrn_measured_fraction_theta_max .NE. *_full          ! Check
PLAT082_ALERT_2_C High R1 Value ................................       0.11 Report
PLAT084_ALERT_3_C High wR2 Value (i.e. > 0.25) ...................       0.33 Report
PLAT088_ALERT_3_C Poor Data / Parameter Ratio ....................       9.41 Note
PLAT220_ALERT_2_C Non-Solvent  Resd 1  C   Ueq(max)/Ueq(min) Range        3.4 Ratio
PLAT234_ALERT_4_C Large Hirshfeld Difference C23      --C25              0.17 Ang.
PLAT242_ALERT_2_C Low    'MainMol' Ueq as Compared to Neighbors of        C16 Check
PLAT242_ALERT_2_C Low    'MainMol' Ueq as Compared to Neighbors of        C23 Check
PLAT243_ALERT_4_C High   'Solvent' Ueq as Compared to Neighbors of        C32 Check
PLAT243_ALERT_4_C High   'Solvent' Ueq as Compared to Neighbors of        C33 Check
PLAT243_ALERT_4_C High   'Solvent' Ueq as Compared to Neighbors of        C35 Check
PLAT244_ALERT_4_C Low    'Solvent' Ueq as Compared to Neighbors of        C34 Check
PLAT360_ALERT_2_C Short  C(sp3)-C(sp3) Bond  C32      - C33      .       1.42 Ang.
PLAT412_ALERT_2_C Short Intra XH3 .. XHn   H25A     ..H26D      ..       1.85 Ang.
PLAT412_ALERT_2_C Short Intra XH3 .. XHn   H34B     ..H36B      ..       1.81 Ang.
PLAT906_ALERT_3_C Large K Value in the Analysis of Variance ......     31.969 Check
```

Alert level G

```
PLAT002_ALERT_2_G Number of Distance or Angle Restraints on AtSite          6 Note
PLAT072_ALERT_2_G SHELXL First  Parameter in WGHT  Unusually Large       0.20 Report
PLAT172_ALERT_4_G The CIF-Embedded .res File Contains DFIX Records          2 Report
PLAT301_ALERT_3_G Main Residue  Disorder .............(Resd  1  )        13% Note
PLAT860_ALERT_3_G Number of Least-Squares Restraints ............           6 Note
PLAT950_ALERT_5_G Calculated (ThMax) and CIF-Reported Hmax Differ           3 Units
PLAT956_ALERT_1_G Calculated (ThMax) and Actual (FCF) Hmax Differ           3 Units
PLAT978_ALERT_2_G Number C-C Bonds with Positive Residual Density.          2 Info
```

```
   0 ALERT level A = Most likely a serious problem - resolve or explain
   5 ALERT level B = A potentially serious problem, consider carefully
  17 ALERT level C = Check. Ensure it is not caused by an omission or oversight
   8 ALERT level G = General information/check it is not something unexpected

   2 ALERT type 1 CIF construction/syntax error, inconsistent or missing data
  11 ALERT type 2 Indicator that the structure model may be wrong or deficient
   9 ALERT type 3 Indicator that the structure quality may be low
   7 ALERT type 4 Improvement, methodology, query or suggestion
   1 ALERT type 5 Informative message, check
```

It is advisable to attempt to resolve as many as possible of the alerts in all categories. Often the minor alerts point to easily fixed oversights, errors and omissions in your CIF or refinement strategy, so attention to these fine details can be worthwhile. In order to resolve some of the more serious problems it may be necessary to carry out additional measurements or structure refinements. However, the purpose of your study may justify the reported deviations and the more serious of these should normally be commented upon in the discussion or experimental section of a paper or in the "special_details" fields of the CIF. checkCIF was carefully designed to identify outliers and unusual parameters, but every test has its limitations and alerts that are not important in a particular case may appear. Conversely, the absence of alerts does not guarantee there are no aspects of the results needing attention. It is up to the individual to critically assess their own results and, if necessary, seek expert advice.

Publication of your CIF in IUCr journals

A basic structural check has been run on your CIF. These basic checks will be run on all CIFs submitted for publication in IUCr journals (*Acta Crystallographica*, *Journal of Applied Crystallography*, *Journal of Synchrotron Radiation*); however, if you intend to submit to *Acta Crystallographica Section C* or *E* or *IUCrData*, you should make sure that full publication checks are run on the final version of your CIF prior to submission.

Publication of your CIF in other journals

Please refer to the *Notes for Authors* of the relevant journal for any special instructions relating to CIF submission.

PLATON version of 13/12/2017; check.def file version of 12/12/2017

Datablock MK460_0m - ellipsoid plot

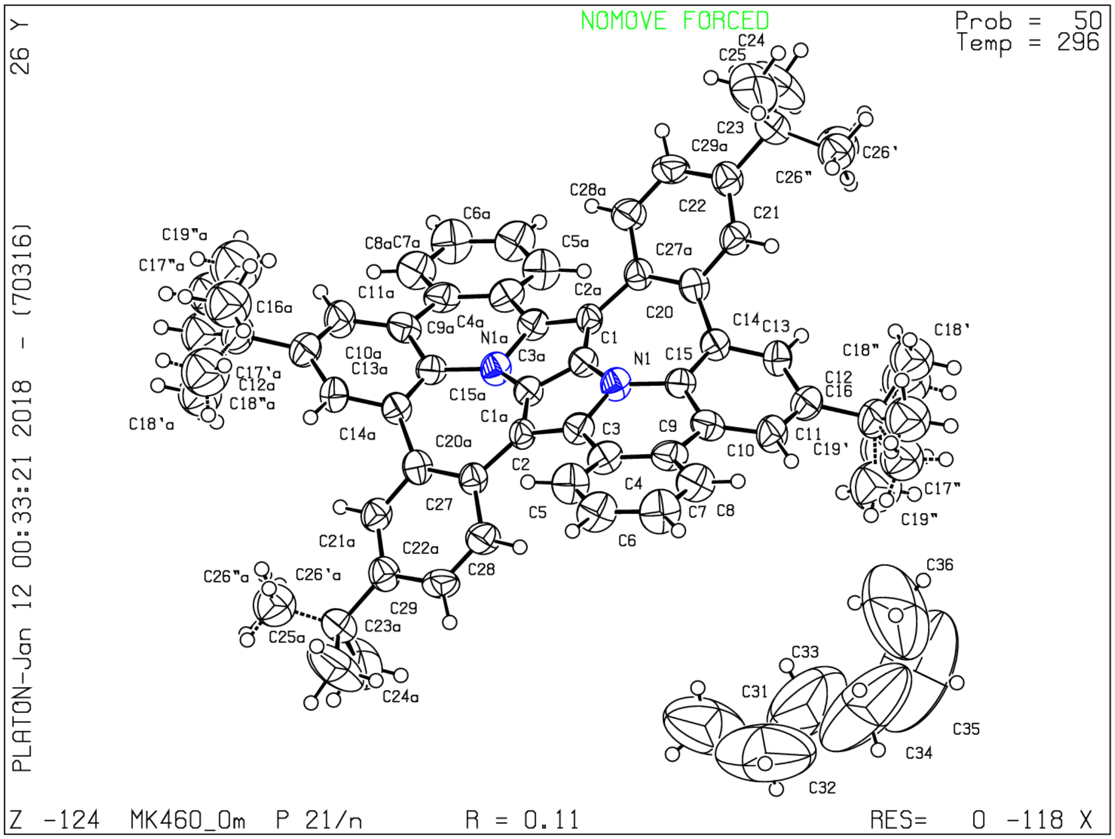

Supplement: Supplementary file 5 — Supplementary Data 3 [file 41467_2018_4144_MOESM5_ESM.pdf]
